# Deconvolution of Blood Microarray Data Identifies Cellular Activation Patterns in Systemic Lupus Erythematosus

**DOI:** 10.1371/journal.pone.0006098

**Published:** 2009-07-01

**Authors:** Alexander R. Abbas, Kristen Wolslegel, Dhaya Seshasayee, Zora Modrusan, Hilary F. Clark

**Affiliations:** 1 Department of Bioinformatics, Genentech, Inc., South San Francisco, California, United States of America; 2 Department of Immunology, Genentech, Inc., South San Francisco, California, United States of America; 3 Department of Molecular Biology, Genentech, Inc., South San Francisco, California, United States of America; Duke-NUS Graduate Medical School, Singapore

## Abstract

Systemic Lupus Erythematosus (SLE) is a systemic autoimmune disease with a complex spectrum of cellular and molecular characteristics including several dramatic changes in the populations of peripheral leukocytes. These changes include general leukopenia, activation of B and T cells, and maturation of granulocytes. The manifestation of SLE in peripheral blood is central to the disease but is incompletely understood. A technique for rigorously characterizing changes in mixed populations of cells, microarray expression deconvolution, has been applied to several areas of biology but not to SLE or to blood. Here we demonstrate that microarray expression deconvolution accurately quantifies the constituents of real blood samples and mixtures of immune-derived cell lines. We characterize a broad spectrum of peripheral leukocyte cell types and states in SLE to uncover novel patterns including: specific activation of NK and T helper lymphocytes, relationships of these patterns to each other, and correlations to clinical variables and measures. The expansion and activation of monocytes, NK cells, and T helper cells in SLE at least partly underlie this disease's prominent interferon signature. These and other patterns of leukocyte dynamics uncovered here correlate with disease severity and treatment, suggest potential new treatments, and extend our understanding of lupus pathology as a complex autoimmune disease involving many arms of the immune system.

## Introduction

Systemic Lupus Erythematosus (SLE) is a systemic autoimmune disease marked by inflammation and tissue damage in multiple organs. Numerous cellular abnormalities have been identified in SLE, including lymphopenia [1], differentiation of dendritic cells [2], and reduced presence of macrophages [3]. Additionally, there are numerous known molecular changes, including increased abundance of TRAIL (Entrez Gene ID 8743) [4], a type I interferon signature[2], [5], [6] and a granulopoeisis signature [6]. Genes affecting risk of developing SLE include IRF5 (Entrez Gene ID 3663), an interferon responsive gene[7], [8] and STAT4 (Entrez Gene ID 6775), a regulator of T-helper 1 cells [9], suggesting that the regulation of differentiation and activation of immune cell subsets is a fundamental component of the disease. All of these cellular, molecular and genetic factors play roles in the disease, but their relative contributions to disease onset and progression are not yet understood. Characterization of the dynamics of SLE at the cellular and molecular level is ongoing and promises to provide insight into the etiology of the disease.

The capability of gene expression microarrays to simultaneously measure essentially all human genes has made possible a variety of approaches to analyzing biological samples [10]. A simple approach is to measure the statistical significance of differentially expressed genes between two groups of samples studied (i.e. patients and controls). This supervised analysis presumes that any meaningful differences are between the predetermined groups of samples. An unsupervised analysis uses no prior knowledge about how the samples are related. As an example, global hierarchical clustering was used to discover the interferon signature in the blood of some but not all SLE patients [2], [5], [6]. Closer integration of biological knowledge of genes with the analysis of expression data can enable more detailed examination of the patient samples. Gene Set Enrichment Analysis (GSEA) is a knowledge-based method to identify genes differentially expressed that share common biological functions or are in the same biochemical pathways [11]. This type of analysis with sets of genes that are specifically expressed in different immune cell subsets[12] can be used to identify the presence of these subsets in disease blood or tissue (data not shown). However, the results are only qualitative, and systematic analysis of relative proportions or activation states of these subsets is not possible by this method.

Microarray expression deconvolution, however, can quantify proportions of cells in a complex tissue. The object of deconvolution is to find the solution of a convolution equation of the form:
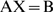
In this case, B is the microarray data from one complex biological sample, X is the set of unknown proportions of the cellular constituents of B, and A is the known matrix of expression levels of the genes in all the cellular constituents of B, which is convolved with X. This matrix equation incorporates gene expression signatures representing cell types (e.g. white blood cells) so it may be solved using standard linear least-squares fitting for X, which is the relative amounts of those cells in the mixture of interest B (e.g. a white blood cell sample). Lu et al. [13] pioneered the application of this technique to microarray data by quantifying the proportions of cells in different phases of the cell cycle in yeast cultures. The deconvolution based on synchronized populations of yeast cells at specific points of the cell cycle predicted the phases occupied by different cell cycle mutants. In another application, Wang et al. [14] analyzed mouse mammary tissue and used the residuals of their fit to separate the differential expression due to changes in tissue composition from those due to intrinsic gene regulation. In both these studies expression signatures of homogeneous samples of cells (i.e. prior biological knowledge) enabled the interpretation of the cellular composition of a complex tissue.

A biological sample from a patient with an autoimmune disease typically contains various different immune cell subsets, and the process of microarray deconvolution can quantify their relative proportions. Essentially, the expression of each gene in the sample is modeled as a linear combination of the expression of that gene in each of the cells comprising that sample. If the expression signature of each immune cell subset is known, then the fractions of each subset in the sample can be determined by solving a linear equation to best fit the fractions of cell subsets to the whole sample's expression signature.

This first step of experimentally determining the signatures of the constituent parts is critical because it defines the framework of the results of deconvolution. The different cell types present in blood can be purified in order to construct expression signatures to use as bases for analysis. We have previously reported the purification and microarray analysis of a large collection of white blood cells (Immune Response *In Silico*, or “IRIS”) [12]. These data include expression of genes in different activation and differentiation states that represent a spectrum of cell species present in blood, providing a basis set for microarray deconvolution of blood samples. Here we test fifteen cell subsets including several resting and activated dyads. Some are not readily distinguishable based on surface markers alone. Moreover, it should be possible to distinguish even greater numbers of cell types by deconvolution.

The expression signatures in blood samples from SLE patients show significant, specific differences from those of healthy controls [6], [15]. Some of these differences are changes in the abundance of specific leukocyte populations [16], [17], suggesting that systematic large-scale characterization of the cellular composition of SLE patient blood would measure quantitative differences relevant to the disease pathophysiology. Here we use microarray deconvolution to explore immune cell subsets and activation states in SLE patient blood. First, we measure the accuracy of the method with a “truth” experiment where known proportions of immune cells are mixed, assayed on expression microarrays, and computationally separated. Next, we performed a proof of concept experiment by deconvolving white blood cell profiles into a modest number of immune cell subsets. We then use this validated method to derive immune cell signatures for a panel of eighteen major populations and states of white blood cells. Finally, we deconvolve expression profiles of blood samples from healthy donors and SLE patients into the proportions of these different white blood cell subsets and identify patterns in their dynamics related to disease and treatment.

## Results

### Establishment and characterization of the deconvolution process

The process of deconvolving mixtures of cells was developed using a system of four transformed cell lines of immune origin: Raji, IM-9 (both from B cells), Jurkat (from T cells), and THP-1 (from monocyte) cells. These cell lines provided the abundant sources of pure cells necessary to support experimental mixing of different types of cells in several different ratios. These cell lines are useful because they show similar but distinguishable expression profiles; their immune derivation is not important to the purpose of the experiment. We chose two B cell lines to gauge the ability of the assay to discriminate between cells that are very similar to each other. The algorithm was trained and the performance limits of deconvolution were measured by creating various mixtures of cells, assaying the pure cells and the cell mixtures on expression microarrays, and using the expression data from the pure cells to deconvolve the expression data from the cell mixtures.

Data for many probesets in a given expression microarray dataset are comprised of noise but little or no biological signal. Here we show that reducing the contribution of these noise-dominant probesets to deconvolution improves performance, and we establish an approach for weighting probesets to define a high-performance basis matrix for performing deconvolution. Probesets were ranked by their degree of differential expression as described in the Methods section, and a thorough set of matrices comprised of different quantities of the most differentially-expressed probesets was tested in deconvolution by comparing the results of each matrix to the known mixture ratios. Both small and very large matrices performed poorly. The distribution of matrix size to the least squares fit to the data was continuous and exhibited a gently rounded optimum at 275 probesets. We observed that goodness of fit correlated very closely with how well conditioned (i.e. condition number) each matrix was. Condition number is a scale-free empirical property of a matrix and a high-fidelity marker for the ability of a basis matrix to accurately deconvolve a mixture. Therefore, subsequent basis matrices were defined by weighting probesets to maximize conditioning.

**Figure 1:**
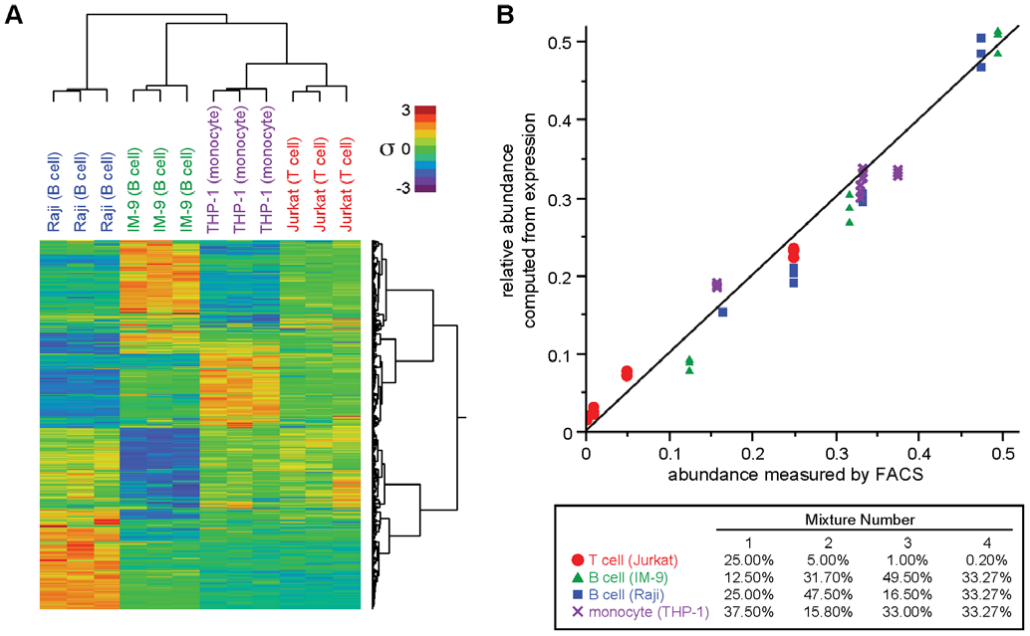
Expression deconvolution was characterized using mixtures of cell lines. (a) Two-dimensional hierarchical clustering of pure cell line samples and the probesets used as bases in deconvolution show strong segregation of cell lines and clear patterns to support deconvolution. (b) Plotting of proportions of cell lines determined from deconvolution vs. proportions of the cell lines actually mixed shows strong congruence (RMS error = 0.028).

Hierarchical clustering of the basis data revealed similar expression signatures within each cell line and very different expression signatures between the cell lines (Figure 1A). These characteristics are not surprising since the approach to defining the basis matrix was designed to maximize them, but it does confirm that there are hundreds of expression profiles that are individually somewhat noisy but together differentiate cell types, and it suggests that mixtures of the cell lines could be deconvolved.

Mixtures of the cell lines were created in defined proportions in triplicate, and each mixture sample was assayed on expression microarrays and computationally deconvolved into its ingredient cell lines. Comparing the resulting predicted proportions to the actual proportions (Figure 1B) showed that the precision of the deconvolution predictions (s.d. = 0.78±0.52%) is sufficient to discriminate immune cell subsets present in proportions below 1% in white blood cell samples. The accuracy of deconvolution (bias = 2.4±1.4%) is comfortably within an acceptable limit of 10%. Neither precision nor accuracy correlated significantly with the concentration of the particular component measured, indicating that the major source of error is additive and not multiplicative.

The variance between the replicates from each cell line was relatively small (s.d. = 0.78%). The two B cell lines, IM-9 and Raji cells, were found by deconvolution to exhibit gene expression profiles that were relatively similar (0.96 mean correlation coefficient). Results from deconvolution of these two cell lines were precise (s.d. = 0.99±0.66%) and accurate (bias = 0.27±1.6%). The difference between the expected and the predicted proportions of B and T cell lines was less precise (average s.d. = 2.4%). It was expected that T cell lines would be more difficult to predict accurately in these test mixtures than B cell lines because the quantities of T cell lines in the mixtures are lower. Surprisingly, the magnitudes and directions of each cell type's errors were not consistent. So although there appears to be systematic error, it is relatively small and not necessarily explained by the cell type.

This characterization of performance on a test data set designed to simulate the challenges of deconvolving leukocytes provides important knowledge of the capabilities of the method that guide its application to whole blood.

### Proof of concept: deconvolution accurately predicts fractions of immune cells in blood

**Figure 2:**
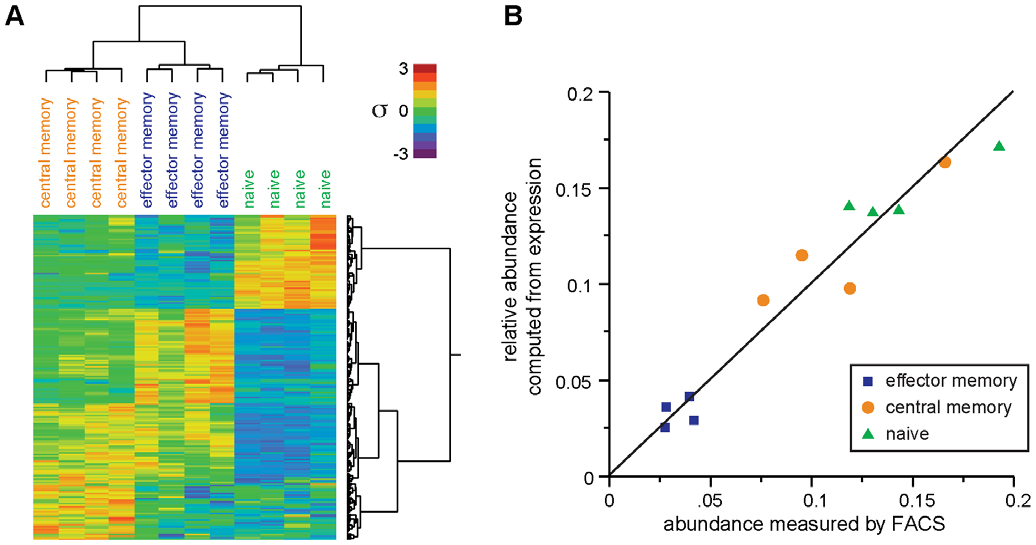
Deconvolution performance was confirmed using T cell subsets. (a) Two-dimensional hierarchical clustering of PBMC and purified T cell samples and the probesets used as bases in deconvolution. (b) Proportions of T cell subsets determined by deconvolution are similar (RMS error = 0.0138) to proportions determined by fluorescence-activated cell sorting.

Next we analyzed blood samples of known composition with several goals in mind: first, to test the method on data independent from that used for training; second, to verify biological applicability of deconvolution by testing performance on leukocytes instead of transformed cell lines; and third, to assess whether deconvolution of white blood cell microarray expression data from healthy donors would correlate well with protein expression of markers that identify immune cell subsets. As a preliminary study, we deconvolved whole blood samples from healthy donors and compared those results to Complete Blood Counts (CBC) with differential of lymphocytes, granulocytes (Figure S1). Although the results were excellent, the CBC data lacked any detailed immune cell subsets to more thoroughly test the performance of deconvolution. Therefore we performed an experiment to test the deconvolution of cell types that are more similar to each other than those in the preliminary CBC study (and more biologically relevant than the pilot experiment that used mixtures of cell lines). We purified naïve, effector memory and central memory T cells from peripheral blood mononuclear cells (PBMC) and compared the quantification of the T cell subsets by expression microarray deconvolution to their levels determined by cell sorting (FACS). Hierarchical clustering of the basis expression data (Figure 2A) illustrates the significant expression differences between the three-subset groups, a necessary condition for the data to permit deconvolution. Many of the genes represented are general markers for T cells, like CD5 (Entrez Gene ID 921) and CD6 (Entrez Gene ID 923). Others like PTK2 (Entrez Gene ID 5747) and SCML1 (Entrez Gene ID 6322) have significantly higher expression in naïve T cells, while CFHR1 (Entrez Gene ID 3078) and MEOX1 (Entrez Gene ID 4222) are examples of genes with higher expression in central memory T cells. Expression deconvolution yielded accurate estimates of the fractions of T cell subsets determined by FACS counting (Figure 2B). These results varied from the FACS data by a mean of 1.3%, illustrating that deconvolution of expression microarray data accurately determines the levels of memory and naïve subsets of T lymphocytes in blood samples.

### Identification of basis probesets to define immune cell subset expression

The proof of concept experiment tested a relatively small number of immune cell subsets. Many others are of interest in an immune disease such as SLE. Microarray data from the immune cell subsets of interest have been reported previously[12] and are summarized in Table 1.

**Table 1:** Leukocyte types used as the basis for whole blood deconvolution. Full description of methods used is published [12].

Name	Methods Summary
Resting helper T cells	RosetteSep CD4+ T-cell enrichment cocktail
Activated helper T cells	Plate-bound anti-CD3 and anti-CD28
Resting cytotoxic T cells	RosetteSep CD8+ T-cell enrichment cocktail
Activated cytotoxic T cells	Plate-bound anti-CD3 and anti-CD28
Resting B cells	MACS CD138 microbeads and CD19 microbeads
Activated B cells	Anti-CD40 and IL4, 23 hours
BCR-ligated B cells	Anti-IgM, 24 hours
IgM memory B cells	sorted CD19+/CD27+/IgG/A−
IgA/IgG memory B cells	sorted CD19+/CD27+/IgM−
Plasma cells	MACS CD138 microbeads and FACS
Resting NK cells	RosetteSep NK-cell enrichment cocktail plus CD2 microbeads
Activated NK cells	IL2, 16 hours
Monocytes	MACS CD14 microbeads
Activated Monocytes	LPS, 24 hours
Macrophages	Differentiated in DMEM culture from monocytes, 7 days
Resting dendritic cells	Differentiated from monocytes with IL4 and GMCSF
Activated dendritic cells	LPS, 24 hours
Neutrophils	Ficoll gradient centrifugation of heparanized blood

**Figure 3:**
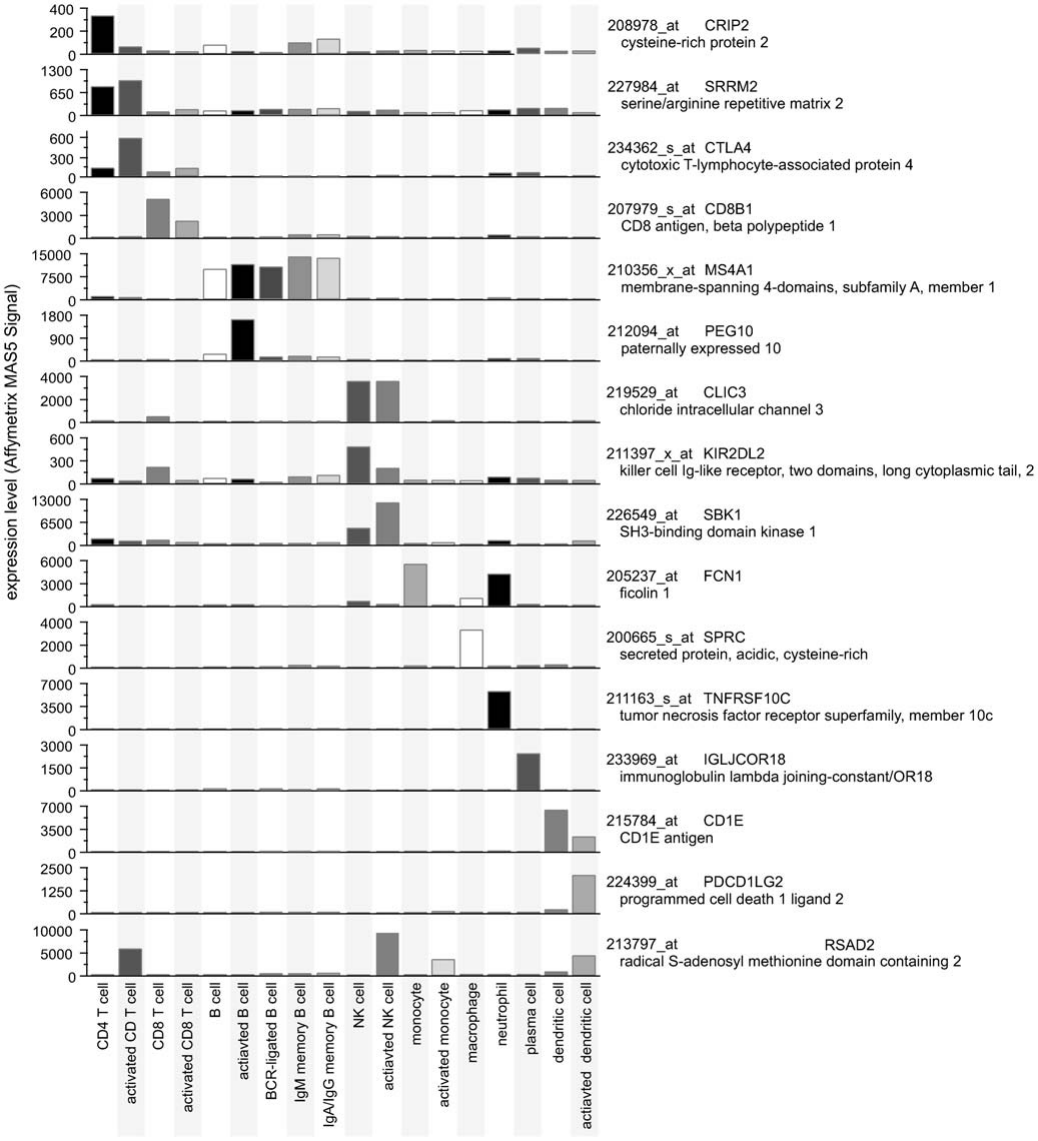
Expression profiles of exemplar probesets in surveyed cell types show strong differences. Microarray expression data for selected basis probesets illustrate expression differences between immune cell types that enable expression deconvolution. Selection of probesets was performed manually to highlight the varying specificity of different genes for different cell types. Data plotted is the mean expression signal of at least three biological replicates. Complete data are available in Table S1.

We selected probesets to use as the basis of discriminating between cell types by screening for those that offered the most significant differences between the several cells in which they were most highly expressed. In order to optimize the number of markers selected, we computed the condition number of matrices of all sizes, from a handful of genes in one extreme, to the whole genome in the other. We observed that the optimal set size was 360 probesets, and we used this set to distinguish between different immune cell subsets and activation states in all subsequent analysis of blood samples. Figure 3 shows some examples of these probesets that discriminate between cell types and are used in deconvolution. Most of these exemplify markers that are relatively specific for one or two cell types. The full collection of basis probesets and their expression levels in all cell types and states are in Table S1. We surveyed the distribution of these data by performing two-dimensional hierarchical clustering and visualized the results as a heatmap with distance-measure dendrograms (Figure S2), and found that the cells all appeared to have distinct expression signatures, to be separated reasonably well on the dendrogram, and to cluster near other samples that we expected to have relatively similar signatures. We examined quantitatively whether the eighteen cell types that we profiled are sufficiently distinct to be resolved by their expression signatures by performing singular value decomposition (SVD) on the basis matrix and observing the values of the diagonal matrix. This method would yield values at the lower-right corner of the matrix near zero if some of the cells were inadequately different from each other; reassuringly, here the lowest value was 3702.301. Although this value is not considered to be near zero and thus not worrisome, it does represent the aspect of white blood cell biology that we had least successfully resolved, so we explored which cells caused it. We noted that the two memory B cell samples were the two samples that were most similar to each other and we hypothesized that they alone might be responsible for the low end of the SVD diagonal. When we tested this by removing the IgM memory population from the basis matrix and refactoring it we found that the diagonal very closely resembled the previous diagonal but with the lowest value missing (Figure S3), confirming that all the cells have been sufficiently differentiated and that the two memory B cell populations are the least differentiated.

The probesets in the basis set represent many genes that are already known to be good markers for particular cell types and states. For instance, well-known B cell markers like CD19 (Entrez Gene ID 930) and MS4A1 (Entrez Gene ID 931) were observed to provide the best discrimination for B cell levels, and CD3 genes like CD3D (Entrez Gene ID 915) were among the best markers for T cells. In some cases the most distinguishing markers were not the classic markers used for cell sorting. For example, it was not surprising that the immunoglobulin heavy chain mu constant region is the most distinguishing marker in microarray data for plasma cells circulating in the blood, but CKLFSF2 (Entrez Gene ID 146225) expression emerged as a superior marker for neutrophils and CLIC3 (Entrez Gene ID 9022) for NK cells.

### Deconvolution of immune cells purified from whole blood

**Figure 4:**
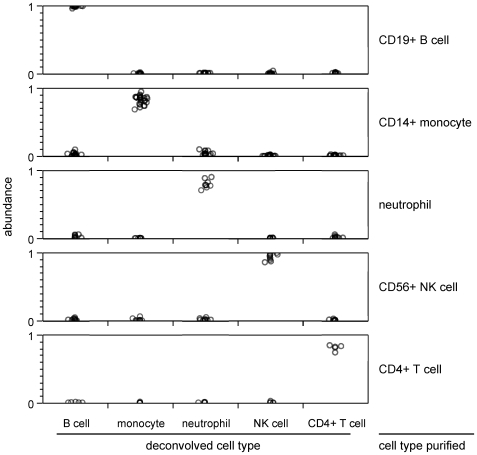
Performance of expression deconvolution on purified leukocytes supports using it on whole blood. Purification and expression deconvolution of an independent test set of leukocytes from whole blood demonstrates that various cell types are properly deconvolved. Plotted data is the calculated fraction of that cell type in the whole sample produced by deconvolution of each of the five purified cell types. Data points are each from different donors.

Validity of deconvolution of whole blood using this large basis set was assessed by experimenting with purified leukocyte samples completely distinct from the samples and data used to build the basis expression matrix. This separate microarray dataset from CD4+ T cells, CD19+ B cells, CD56+ NK cells, CD14+ monocytes, and neutrophils purified from different donors were deconvolved and the results are shown in Figure 4. Results from resting and activated cell populations were added to yield total cell abundance for each cell type where appropriate. Each purified cell type was determined to be mostly pure and contaminated by less than 10% of each other cell type, confirming the validity of the basis set and its application to blood deconvolution.

### Deconvolution of SLE patient and healthy donor blood

**Figure 5:**
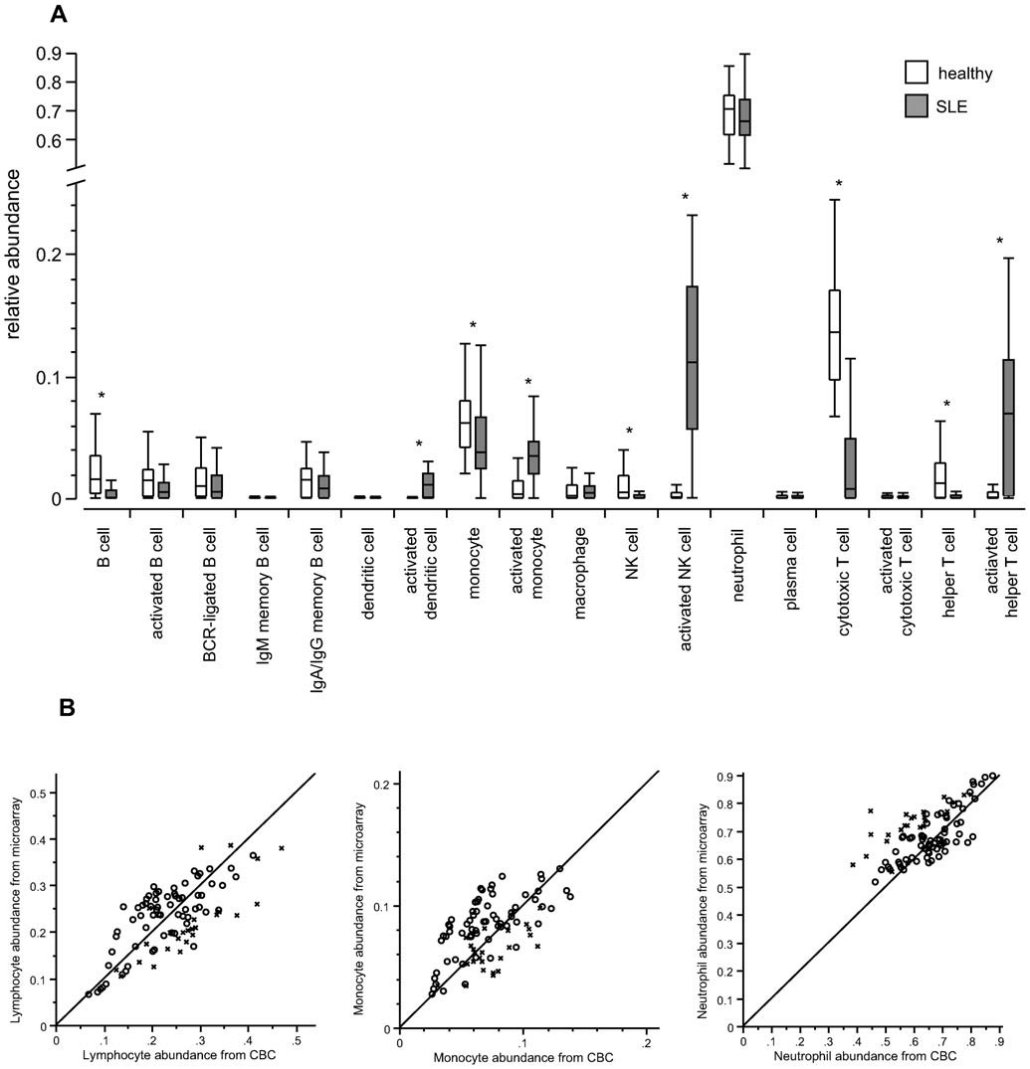
Complete leukocyte deconvolution of healthy or SLE whole blood shows significant differences. (a) Deconvolved relative abundance of different leukocyte cell types and activation states exhibit statistically significant differences between healthy donors and SLE patients in many (noted by “*”; p-values are 8.6e-07, 3.1e-10, 3.5e-04, 1.5e-09, 1,1e-03, 1.6e-15, 2.2e-16, 6.9e-06, 1.1e-08, respectively). Quantile boxes and tails are 10%ile, 25%ile, 50%ile, 75%ile, and 90%ile. (b) Determination in healthy or SLE blood of relative abundance of total lymphocytes, monocytes, or neutrophils by CBC differential compared to determination of relative abundance by deconvolution. Diagonal lines are y = x, shown for reference, highlight the agreement between the two methods.

With the method of deconvolution validated and characterized and a rich set of probesets and cell types defined for use in blood, we investigated samples of biological interest for which the relative abundance of cell types was unknown. Deconvolution was performed on microarray expression data from white blood cell samples from 72 SLE patients and 45 healthy donors (Table S2). Proportions of cells of each type and state were calculated (Figure 5A). Residuals from fitting the model were relatively low, with a median of 0.11. Stability of the fits was analyzed by repeating the fitting process with each of various cell types omitted (i.e. “leave one out” testing); we observed that there was only a very small disturbance in the results of the fit by the remaining cells. Furthermore, the disturbance that did occur was confined to cells that were very similar to the one that was omitted (Figure S4). Overall SLE patients displayed mild lymphopenia: for T, B, and NK cells the sums of all cell counts of each cell type was found to be significantly lower in SLE than in healthy controls. In order to compare the degree of activation of different leukocyte types we define activation as the ratio of the abundance of activated cells of a given type to the abundance of resting cells of that type. T helper cells, NK cells, and monocytes were each found to be significantly activated in SLE compared to healthy controls. NK cells were on average 149-fold more activated in SLE, while T helper cells were 59-fold more activated. In contrast, B cells and cytotoxic T cells displayed no significant activation. Dendritic cells were undetectable (at or below the lower limit of detection) except for activated dendritic cells in SLE. Although their activation cannot be accurately calculated using the definition of activation used here, the abundance of activated dendritic cells at almost 2% in SLE samples is significant and notable. Neutrophil and plasma cell levels were not significantly different between SLE patients and healthy controls.

**Figure 6:**
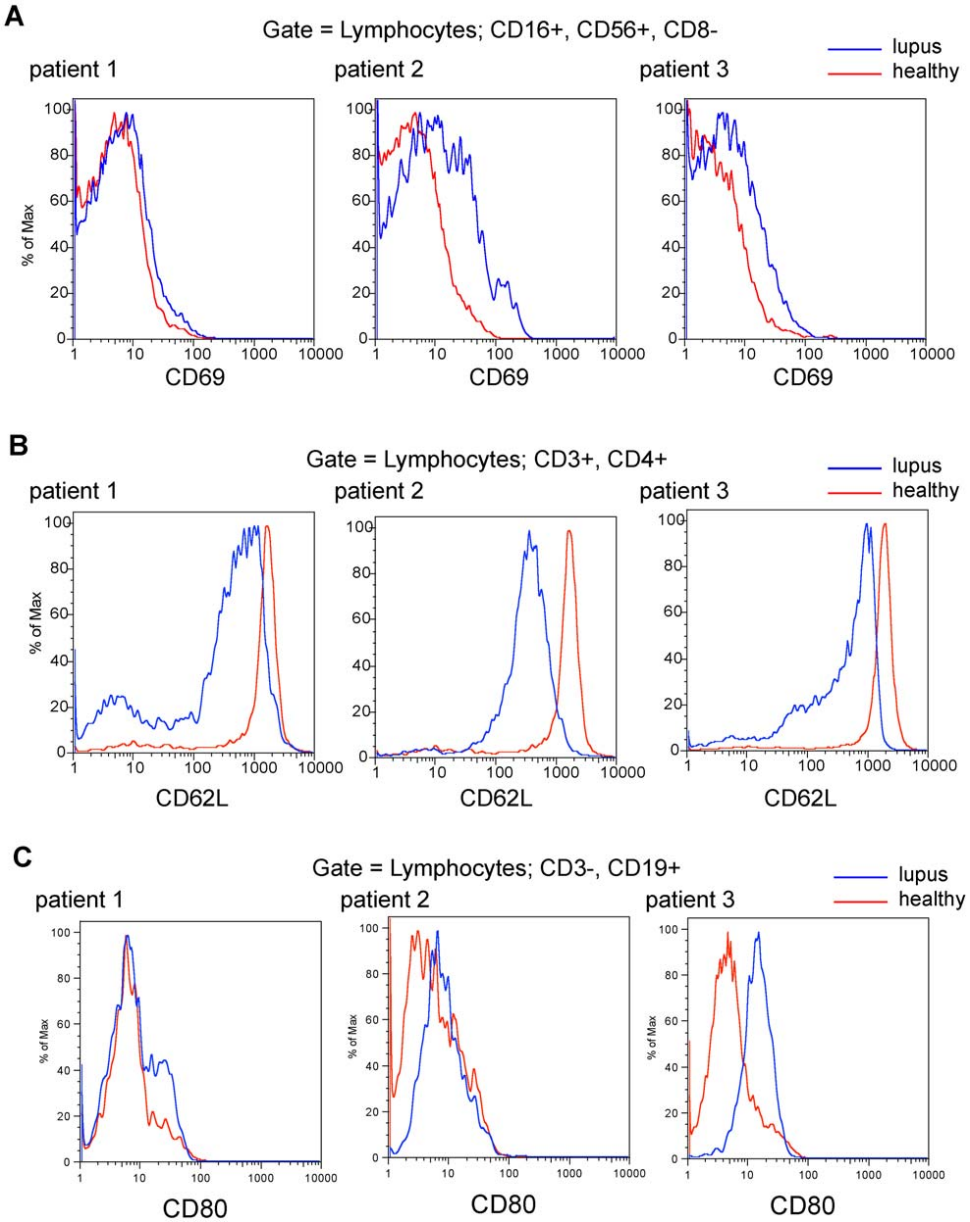
FACS counting validates key findings from expression deconvolution. Quantifying levels of resting or activated lymphocytes from a separate validation cohort by purifying and staining with markers of activation validates the two most significant findings from microarray deconvolution of healthy and SLE patients' blood. (a) NK cells purified and stained for the classical NK activation marker CD62 show significant activation in two patients and mild trend towards activation in a third. (b) CD4+ T helper cells purified and stained for the marker of naïve T cells CD62L show significant downregulation, indicating activation and suggesting a transition to a memory phenotype. (c) CD19+ B cells purified and stained for the marker of activated B cells CD80 show mixed results: no change in one patient, mild upregulation in another, and strong upregulation in the third.

We tested these results' validity by comparing them to counts of total lymphocytes, total monocytes, and neutrophils determined by CBC differentials performed on the same blood samples (Figure 5B). The correlations between the two methods were reasonably good (average Pearson coefficient = 0.5196) and very consistent between different cell types (standard deviation of the mean = 0.136). To specifically validate findings of particular interest from deconvolution, we purified and quantified activated NK and T helper cells from SLE patients and healthy controls and tested their level of activation using the conventional method of FACS. Purified NK cells were stained with the classical activation marker CD62L. Consistent with results from deconvolution, two out of three patients showed substantial activation of NK cells compared to healthy donors, while one patient showed mildly elevated levels of NK cell activation (Figure 6A). CD4+ T helper cells purified and stained for the marker of naïve T cells CD62L show significant down-regulation (Figure 6B), indicating activation and suggesting a transition to a memory phenotype. To validate our unexpected finding that B cells were not activated in lupus patients we purified B cells from the same three patients and quantified levels of CD80, a molecule found on the surface of activated B cells that provides a costimulatory signal for T cell activation. Two of the three patients' B cells possessed increased levels of CD80 (Figure 6C). To reconcile this observation with the lack of B cell activation in the main SLE cohort we examined CD80 levels in the expression profiles of the reference resting and activated B cell samples used for deconvolution and found no significant difference between the two groups.

**Figure 7:**
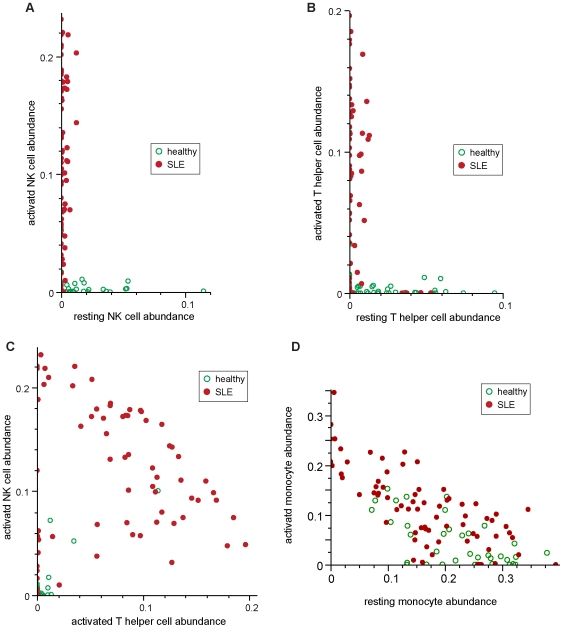
Significant relationships exist between activation patterns of different cell populations. Visual analysis of T or NK cell types' abundance from individual donors of the main cohort reveals further patterns. (a) Plotting of resting vs. activated NK cells shows that activation of NK cells occurs to all NK cells simultaneously. (b) Plotting of resting vs. activated T helper cells shows that likewise activation of T cells occurs to all T cells simultaneously. (c) Plotting of activated NK cells vs. activated T helper cells shows that most patients' populations of these two cells appear to lie along a negatively sloped line. Linear least squares fitting to those samples yields a line of slope −1.00078. (d) Levels of resting and activated monocytes show a negative relationship with each other in both SLE and healthy donors.

Next we investigated whether there were specific relationships between the quantities of pairs of different immune cell compartments by examining the distribution of each pair of immune cells in healthy and SLE individuals visually. Levels of T helper cells and NK cells vary widely in both healthy and lupus blood samples. However, there are no cases where moderate or higher levels of both resting and activated cells exist in the same sample (Figure 7A and B). Either most of these lymphocytes are activated or very few are activated. Most of the SLE patients appear to have either a greater proportion of activated T helper cells or a greater proportion of activated NK cells than do healthy individuals (Figure 7C). Additionally, most patients seem to have a similar number of activated cells. In some patients there are more activated T cells, and in others there are more activated NK cells. This observation suggests that the total number of activated T helper and NK cells is regulated. K-means clustering assigned patients in this study into three discrete groups: those with a high proportion of activated T cells, those with a high proportion of activated NK cells, and those with low activation in both immune cell subsets. Linear least-squares fitting on the two groups with a high proportion of activated cells measures the hypothesized maximum activation state to have a slope of negative one (slope of −1.00078, RMS error of 0.044). This supports the model of a regulated activation state in lupus patients that can be achieved either through activation of T cells or NK cells. Comparison of levels of resting and activated monocytes (Figure 7D) showed that total levels of monocytes are roughly constant and that as increasing levels of activated monocytes are observed, decreasing levels of resting monocytes are seen. This relationship is seen in both SLE patients and healthy donors. As noted previously, SLE patients showed a higher average abundance of activated monocytes, although the total monocyte counts in the two groups are comparable.

**Figure 8:**
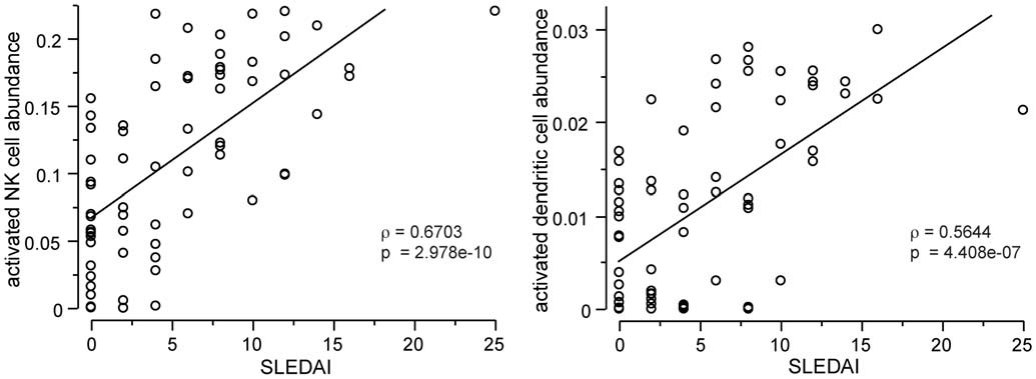
Disease activity is related to changes in cell populations. SLE disease activity index (SLEDAI) scores from the main SLE patient cohort are significantly correlated with activated NK cell or activated dendritic cell relative abundance. Diagonal lines and statistical metrics are from the linear least squares fit to the plotted data.

**Figure 9:**
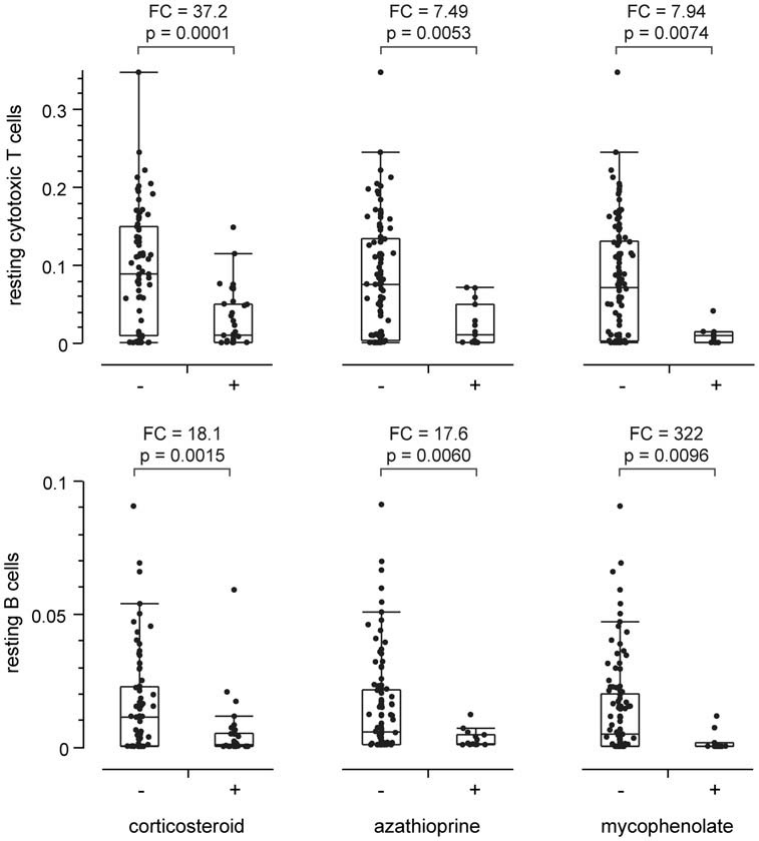
Medical treatment is related to changes in cell populations. Relative abundance of selected cell types for SLE patients from the main cohort segregated by whether the patient was currently on corticosteroid, azathioprine, or mycophenolate treatment. Fold changes shown are fold change of the mean of the data. P-values shown are from two-tailed Wilcoxon Rank Sum test. Quantile boxes and tails are 10%ile, 25%ile, 50%ile, 75%ile, and 90%ile.

To further explore the potential significance of these findings, we next explored whether immune cell levels in the main cohort were related to clinical parameters such as patients' disease activity, medication, or traditional hematological measures. Comparison of the abundance of immune cells with clinical disease severity showed significant positive correlation between patients' SLEDAI (Systemic Lupus Erythematosus Disease Activity Index) scores and the abundances of activated dendritic cells and activated NK cells (Figure 8), but other immune cell types were not significantly correlated with SLEDAI. Complement C3 and C4 levels were compared to immune cell levels and found to be not significantly correlated (data not shown). In order to explore possible relationships between medication and immune cell levels we categorized the SLE patients according to whether they were or were not currently undergoing treatment with corticosteroids, azathioprine, or mycophenolate and then tested for differences in the level of immune cell type based on the presence or absence of each treatment. Populations of resting B cells and resting cytotoxic T cells were both reduced significantly (Figure 9) by each treatment, while the populations of these two cell types in their activated state were not significantly different (data not shown). No other cell types' levels were affected as much by medication, nor were any of them significantly affected by more than one medication class, in contrast to B and CD8 T cells' relationship to all three classes of medication. We also examined whether changes in cell abundance were related to current use of selective serotonin reuptake inhibitors (SSRI), Angiotensin Converting Enzyme (ACE) inhibitors, statins, anti-malarial medications, or beta-blockers and found no significant effects (data not shown).

## Discussion

Microarray deconvolution is an emerging method for measuring proportions of cell types or states in complex systems. The studies reported here are the first application of this technique to human biology, the first application to blood, and the first application to study immune disease. The autoimmune disease SLE is a prime example of a disease where determining the proportions of immune cells is an important contribution to understanding the etiology of the disease. In addition to the biological advances, this study extends previous work on deconvolution in several technical ways that are necessary to support its application to SLE. First, we validate the method using complex biological samples of known composition in order to show that it can be performed on blood samples. Second, we measure the performance of the method on a controlled system with a precisely known answer. Third, we expand the number of component cell types quantified in mixed samples, and we validate the method on an independent test set of those cell types.

Here we find that patients suffering from SLE have some types of blood cells activated predominantly: NK cells have the highest activation, followed by T helper cells, monocytes, and dendritic cells. This activation is validated by FACS analysis of blood samples from an independent cohort of SLE patients and healthy donors. Also, it is consistent with previous observations that genes upregulated in response to interferon signaling are expressed at high levels in lupus patients [6]. Many of these interferon responsive genes are present at increased levels in the activated versus resting lymphocytes observed here, suggesting that NK cells, T helper cells, and monocytes are the circulating cells principally responsive to type I interferon. Type I interferons are also known to promote maturation of monocytes into conventional dendritic cells (cDCs) [18], and this activity is consistent with our observation that activated dendritic cells appear in the blood of SLE patients. CDCs respond to bacterial infections and mediate the associated exacerbations in SLE patients, and they have been found to release IFN-alpha upon TLR4 ligation following priming with IFN-beta [19], consistent with their apparent involvement here.

Conventional methods that use gene expression to quantify biological processes must use a single gene or a set of well-correlated genes, but solving a system of gene expression equations surmounts this limitation. For instance, RSAD2 (shown at the bottom of figure 2) would be a relatively poor cell type marker but is very valuable when quantifying cell types by the methods used here, since it is significantly differentially expressed between many different cell types. This gene is also important here because it is well-established to be induced by type 1 interferons [20]. Among the activated cell types considered here, RSAD2 expression is high in those (and only those) that are found to be more abundant in SLE patients. Therefore, this gene (and others with similar expression profiles, not shown) and the results here based on it support current views that the interferon signature observed in most SLE patients is due to the simultaneous activation of several classes of leukocytes by type 1 interferons and that this class of cytokines is central to the disease (reviewed in [21]).

The dynamics of activation appear to differ among cell types. For instance, monocytes constitute approximately the same fraction of blood in all samples examined, and the distribution of the proportion of resting to activated monocytes was fairly uniform across its observed range. In contrast, both NK and T helper lymphocytes were essentially fully activated or fully resting. Unlike FACS, the algorithm used here cannot differentiate between a population of a given cell type that are all homogeneously mildly activated and a population of that cell type that consists of a mixture of some resting cells and some strongly activated cells, since the parameters used to assess activation are merely the total quantity of each gene's mRNA present in the sample. The monocytes observed here might possess the ability to adopt a mildly activated state, or perhaps they function in discrete activation states like lymphocytes appear to but differ in that the entire population of monocytes need not all be resting or activated in unison.

In some cases the level of activation of a patient's white blood cells was correlated among different cell types. For instance, dendritic cells' and NK cells' levels of activation are positively correlated. However, some types of lymphocytes exhibit more complex negative relationships. The maximum level of activated NK cells and T helper cells in SLE patients appears to be constant at approximately 30%. This is clearly seen in most SLE patients, but healthy individuals typically have substantially fewer activated lymphocytes. Although the results presented here offer no direct evidence about the mechanisms by which this pattern occurs, the data are consistent with the existence of a regulatory system that constrains the maximum total level of these two cell types to 30% and permits shifts in the balance between them. Both activated T helper cells and activated NK cells exhibit signs of being important to SLE pathology [16], [22]–[25], even at the transcriptional level [26]. Perhaps their relative levels define clinically important subtypes of SLE, and if so they might be useful diagnostic markers for this disease characteristic.

Several of the cell types that are hypothesized to be particularly important in SLE are correlated with patients' clinical measurement of disease activity. Activated dendritic cells are efficient antigen presentation machinery, and this cell type is found here to be more abundant in patients with relatively active disease, consistent with correlations between autoantibody blood titer and SLE disease activity [27].

Other immune diseases may be amenable to the methods presented here. One challenge will be the interpretation of predictions in a disease that affects specific organs such as rheumatoid arthritis where there is less evidence of a systemic effect. Deconvolution of biopsies from these patients would have to be interpreted with caution as individual cell types are difficult to isolate from solid tissues for any validation. Moreover, biopsies would contain significant quantities of cells of unknown type. Therefore their signature genes would not be included in the basis matrix for deconvolution. Of course, expanding the arsenal of data on basis cell types would enable thorough analysis of such diverse kinds of samples.

Expression deconvolution has several advantages over cell sorting[28] or other traditional methods for quantification of cell species. First, conventional methods deal with at most a handful of different cell types at one time, while expression analysis can simultaneously quantify a much greater number of cell types. Second, deconvolution integrates the partially redundant information of a large number of genes to yield its results; this redundancy is important because it mitigates the contribution of noise inherent in biological measurements and thus boosts robustness. Third, post-hoc analysis of expression datasets is relatively rapid, and there are a large and growing number of datasets publicly available. Novel methods of expression analysis like deconvolution have the potential to further tap this important resource for the scientific community.

One concern with the application of a system of linear equations such as used here is the mathematical structure of the problem: is it well posed, what is the dimensionality and conditioning of the matrix. The dimensions of the matrix are important because a system of linear equations must be overdetermined- it must have more equations than unknowns in order to not have multiple perfect solutions, and in this case there are 360 equations and only 18 unknowns. The equations must be linearly independent- that is, each sample's expression signature must not be the equal to the sum of other signatures when they are each multiplied by any constant- this requirement is also satisfied. Finally, the matrix must be well conditioned. That is, the solutions yielded must be relatively stable and not overly sensitive to small fluctuations in input data. This characteristic represents a satisfactory bound on the error of the solution for a given mixture, and can be thought of as the result of a basis matrix that contains genes that together form a complete but parsimonious set of robust markers for the cell types of interest. The property of conditioning is quantified as the condition number, a positive scalar value that is low when the matrix is stable. As expected, the condition number was found to be high for small or large numbers of genes and to have a minimum for moderate numbers of genes. The basis matrices used here were constructed in part by minimizing their condition number, and they do in fact have condition numbers near zero. The stability of the whole blood basis matrix was verified by comparing results of deconvolution of whole blood using all cell types to results obtained from deconvolution of the same samples with one or two of the cell types omitted. Omission of a few cell types did not grossly alter the results for the other cell types. Reassuringly, those that were altered were the ones that would be expected. For instance, omitting activated dendritic cells from the basis matrix caused resting dendritic cells to be estimated slightly higher in abundance since those two signatures are substantially similar.

There are several limitations of microarray deconvolution that bear discussion. One is that it produces answers about the composition of a sample only for a given set of constituents, in this case the immune cell subset profiles that form the basis matrix. This set can be constructed beforehand or determined via bootstrap methods from the mixture data itself, but either way it may be incomplete or inaccurate. We have assembled a fairly comprehensive set of immune cell subsets with which to analyze SLE, but there are likely to be important subsets not profiled here. Two cell types in particular are known to play important roles in SLE but are not included here because they were deemed to be too rare in blood to be reliably detected by this expression analysis approach: regulatory T cells and plasmacytoid dendritic cells. Regulatory T cell levels have been found to be higher in SLE patients and to correlate with disease activity [29], and their interactions with NK cells [30] make them a very interesting and relevant cell type. Plasmacytoid dendritic cells are the major source of type 1 interferons and are thought to be central to this axis of SLE (reviewed in [31]). Although technically intractable here, inclusion of these cells in studies of this kind would likely offer further insights into SLE biology. Memory T cells were also omitted from the SLE analysis even though they were profiled in the proof of concept experiment because we chose to subset T cells for the basis matrix by CD4/CD8 status and activation status. Adding yet another orthogonal factor, memory status, would have multiplied the number of T cell profiles from four to twelve, an extension that would be interesting but is beyond the scope of this work.

The conspicuous lack of increase of activated B cells or plasma cells in our survey of SLE samples may be due to dissimilarity between the *in vitro* stimulated B cells and CD138+ purified plasma cells we used and the populations of plasma cells that produce the autoreactive antibodies that are a hallmark of SLE. We performed both canonical cytokine activation of B cells and B Cell Receptor ligation via anti-IgM antibodies, but neither protocol for B cell activation yielded a signature that was detected at a higher level in SLE than healthy controls. We confirm in the validation samples that the level of the activation marker CD80 is elevated on B cells from some SLE patients and show that this marker is not differentially upregulated in our *in vitro* activated B cell cultures. This resolves the apparent conflict between the results presented here and the general view that B cells are activated in SLE disease, and it underscores the importance of capturing the complexity of relevant cell biology as fully as possible in any set of basis samples to be used for expression deconvolution.

Lahdesmaki *et al.* [32] used a Bayesian approach to deconvolution that avoids *a priori* definition of basis groups and instead estimates them. Successful application of this approach requires a dataset with a relatively large number of cell types with linearly independent expression profiles. In our hands, use of the biological knowledge of important immune cell subsets and our definitions of their expression profiles increased the accuracy of deconvolution predictions.

Another limitation of microarray deconvolution is the discrete nature of the component cell types in the basis set. The model assumes that cells do not exist in significant quantities in states intermediate between those that are purified and profiled for inclusion in the basis set. This issue has been addressed in one study: continuous variation in the expression levels of genes have been mapped to continuous cellular states in cell cycle experiments in yeast [33]. Immune cells also exist at different points in a continuum of states of differentiation and activation. However, we did not consider these states here because of the complexity of modeling the large number of intermediates stemming from a group of eighteen basis cell types. We did observe that the residuals from fitting of immune cell basis matrices to the SLE blood in this study were small, indicating that the discrete model's assumptions are valid. We assume that the residuals are due to a combination of technical noise and incomplete sampling of expression profiles of leukocyte populations. The relative contributions of these two factors are not known, but we predict that fit could be further improved if the different states of immune cells were more fully represented. For example, it might be advantageous to capture varying degrees of activation by contrasting signatures of cells that are resting and activated *in vitro*. Also, the canonical activation of cells used in this study are widely believed to simulate *in vivo* activation reasonably well, but there are other forms of activation (e.g. Th1/Th2/Th17/Treg cell polarization) that could be performed and profiled on microarrays to better capture the spectrum of leukocyte populations in blood. Expression deconvolution may actually even have the potential to help identify these cells or states. In this study we selected genes that discriminated between the cell types we had chosen to assay; this step improves the performance of the method but likely implicitly excludes the best markers for cell types that were not chosen. If the approach was modified to include more genes, then the subset of those extra genes that fit poorly might be good markers for cell types or states that should have been profiled but were not. Alternatively, the solution based on an optimal set of basis genes could then be used as a standard against which to measure the fit of each other gene measured on the microarray, and the genes with substantial expression that is under-predicted or not reflected in any of the basis components could be considered a candidate marker for an important cell type or cell state that remains to be profiled. We imagine that the responses of leukocytes to the wide variety of cytokines that they might encounter would be the most prominent discoveries yielded by this approach.

There are other possible causes of residuals. For example, a recent study exploring the heterotypic interactions between human cancer-derived cell lines and stromal fibroblasts[34] finds that a minority of genes show interaction effects. Determining all of these interaction effects is intractable if the number of cell types involved is more than three or four, but nevertheless this issue exists and may be a source of error in a linear model that does not incorporate it. Interestingly, this study used microarray data to explicitly quantify heterotypic interactions between the cancer and stromal cells *in vitro*, and this approach could be applicable to leukocytes.

The deconvolution results reported here are interesting observations of activated states of lymphocytes in SLE patients. This could be confirmed as meaningful to disease progression by extending this analysis to longitudinal data from SLE patients with blood collected at multiple time points. Patients may also respond differently to therapies based on their deconvolution results. We have proposed that there is a regulation of the maximum activation of lymphocytes. Drugs targeting positive or negative regulators of the activation of different lymphocytes may show an effect on the slope or percent of the maximum activation state we observe. In summary, deconvolution can provide a powerful insight into the immune response in patients with autoimmune disease.

## Methods

### Ethics Statement

The collection and analysis of the testing cohort samples was approved by the Institutional Review Board of Gene Logic Corporation and written informed consent was obtained from all patients. The collection and analysis of the validation cohort samples was approved by the University of Michigan internal review board and written informed consent was obtained from all patients.

### Cell lines

Cell line samples were prepared as follows: human cell lines Jurkat (T cell leukemia), THP-1 (acute monocytic leukemia), IM-9 (B lymphoblastoid multiple myeloma) and Raji (Burkitt B-cell lymphoma) were obtained from the American Type Culture Collection (ATCC). Cells were grown in RPMI 1640 medium with 2 nM L-glutamine adjusted to contain 1.5 g/L sodium bicarbonate, 4.5 g/L glucose, 10 mM HEPES, 1 mM sodium pyruvate, and 10% fetal bovine serum. Medium was renewed every 2–3 days and cell density did not exceed 3×106 cells/ml.

### Purification of Leukocyte Populations

Eighteen compartments and activation states of leukocytes were purified and treated as described previously[12] and are summarized in Table 1.

### Clinical samples

T cell subsets for the proof of concept experiment were prepared as follows: peripheral blood mononuclear cells (PBMCs) were isolated from healthy donors via leukopacks as previously described [12]. A portion of each PBMC sample was assayed on gene expression microarrays, while the remainder was used for further purification. PBMCs were enriched for CD4+ T cells using MACS beads for negative selection as per manufacturer's recommendations. CD4+ T cells were then labeled with CCR7-FITC, CD45RO-PE, CD62L-PECy5, CD45RA-APC and CD4-PECy7 and sorted by FACS into the following subsets: naive (CD4+CD45RA+CCR7+CD62L+), central memory (CD4+CD45RO+CCR7+CD62L+) and effector memory (CD4+CD45RO+CCR7−CD62L+/−). FACS profiles of cell sorting are shown in Figure S5.

Pure CD4+ T cells, CD19+ B cells, CD56+ NK cells, and CD14+ monocytes used for confirmation of the deconvolution method were isolated from whole blood obtained from healthy donors by Ficoll gradient centrifugation followed by negative selection. Purity was confirmed by FACS with positive markers.

### Patient selection

Whole white blood cell samples for exploratory analysis were collected from 72 SLE patients and 45 healthy donors (the test cohort) by Gene Logic Corporation (Gaithersburg, MD). Demographic information on this cohort is shown in Table 2. Three additional patients with active SLE were recruited from the outpatient rheumatology clinic and in-patient services at the University of Michigan. These patients, referred to as the validation cohort, were used for FACS confirmation of results obtained from the test cohort. Written informed consent was obtained from all patients. Patients were selected as being over 21 years of age and meeting the diagnostic criteria of the American College of Rheumatology (ACR) for SLE at the time of the visit. Clinical data were collected for each visit and include disease activity as assessed by the SLEDAI activity index, clinical laboratory test results, and current medications.

**Table 2:** Summary of SLE patients and healthy controls studied by microarray deconvolution of whole blood.

	SLE	Healthy Control
Number of patients	72	45
Female/Male	72/0	26/19
Median age at time of sample collection	33	29
Median C3 complement (mg/dl)	135	n.d.
Median C4 complement (mg/dl)	21	n.d.
SLEDAI 25%ile/median/75%ile/max	0/4/8/25	0
Immunosuppressant therapy	15 (21%)	0

### Microarray sample processing

Total white blood cell RNA was obtained using the RNeasy Midi kit and the “RNeasy Midi Protocol for Isolation of Total Cellular RNA from Whole Blood” protocol (Qiagen). Purified leukocyte RNA was obtained using the RNeasy Mini Kit (Qiagen). Manufacturers protocol was followed for all steps and the optional on-column DNase treatment was performed. RNA was quantified using ultraviolet spectrophotometry. RNA was labeled for and hybridized to Affymetrix™ HGU133 expression microarrays assay using standard Affymetrix™ GeneChip™ protocols. Scan data from microarrays was processed by Affymetrix™ Microarray Analysis Suite™ software version 5 to yield Signal data.

### Computational and statistical methods

All statistical and matrix algebra calculations were performed using the R Project software package [35].

Hierarchical clustering was performed on log2-transformed data by hierarchically clustering using Pearson correlation coefficient as the similarity metric and average-linkage for node summarization.

Correlation tests between cell types and SLEDAI scores were Spearman correlations, with p-values obtained using the “cor.test” function. Differential cellular abundance by treatment status was tested by Wilcoxon Rank Sum test on linear data.

Expression deconvolution was performed on linear, untransformed data as follows: in each mixture sample, the total expression signal of each microarray probe was modeled as the sum of the expression signals of its constituent parts, each of which is described as a product of the expression signal of that probe in that purified cell type times the fractional abundance of that cell type in the mixture:
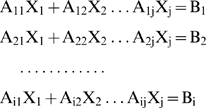
where A_ij_ is an expression signal measurement in a purified cell, B_i_ is an expression signal measurement in a mixture of cells, and X_j_ is a fractional abundance, for each of i probesets and j cell types. These equations may be rewritten as a matrix equation:
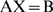
where A is the basis matrix of the expression levels of all probesets in all cell types, B is the vector of expression levels of all probesets in one mixture, and X is the vector of the relative levels of cell types comprising B. The equation was solved for X with the R function ‘lsfit’ (a linear least squares algorithm) followed by removal of the lowest negative coefficient from the equation and iteration of the solution if necessary until all coefficients were non-negative. Fractions of the cell types were determined by dividing the coefficients by the yield of mRNA per cell input.

The probesets comprising the basis for deconvolution were the subset of all probesets that maximized conditioning of the basis matrix. They were determined as follows: top differentially-expressed (based on 95% fold change confidence intervals from Student's T-test) probesets were determined by comparing each probe's highest-expressed group with the next highest-expressed group in order to find probesets that are good markers for each cell population. This step was repeated with comparison between the top group and the third-highest group in order to also include probesets that were strong markers for two cell populations. Basis matrices of increasing numbers of top probesets picked separately from both comparisons and from each group were created and their condition number calculated. The function of condition number based on the number of probesets used to calculate it was observed to be continuous and relatively high for very small or very large numbers of probesets with a minimum for intermediate numbers of probesets (Figure S6). The matrix with the lowest condition number (i.e. the best-conditioned) was selected as the basis matrix to be used for deconvolution. The identities, accession numbers and expression data of probesets in the basis matrix for leukocyte deconvolution are provided in Table S1.

### Accession Codes

GEO: microarray data, GSE11057 and GSE11058.

## Supporting Information

Table S1 Mean expression data for the pure cell types and the probesets used as the basis matrix for performing expression deconvolution on whole blood samples from the main clinical test cohort or on the validation set of purified leukocytes.
(0.11 MB PDF)

Table S2 Expression data for the basis probesets for the main clinical test cohort of SLE patients and healthy controls.
(0.31 MB XLS)

Figure S1 Expression deconvolution is consistent with a CBC differential. Determination in one healthy donor of relative abundance of total monocytes, neutrophils, or lymphocytes by CBC differential compared to determination of relative abundance by deconvolution.
(0.44 MB EPS)

Figure S2 Two-dimensional hierarchical clustering of the mean expression data from purified leukocyte samples for the probesets used as bases in deconvolution shows clustering of similar cell types and sparse distribution of high expression of marker genes.
(1.18 MB EPS)

Figure S3 Singular value decomposition of the basis matrix yields a set of values in the diagonal matrix that are all significantly above zero, indicating that all cell types are linearly independent. Refactoring of the basis matrix following removal of IgM memory B cells yields a set of singular values that are very similar except for the absence of the lowest value. This indicates that this relatively small lowest singular value is caused by the close similarity of the two memory B cell types.
(0.26 MB EPS)

Figure S4 The basis matrix is robust to omission of one or two cell types. Healthy whole blood samples were deconvolved by either a complete basis matrix or by a basis matrix missing (A) activated dendritic cells, (B) activated NK cells, or (C) both IgM and IgG/IgA memory B cells. Each scatterplot compares the complete matrix result with the result from omission of that specific cell type or types and shows how solutions are relatively stable to variations in the input data but variations in the profiling of related cell types do affect each other.
(0.49 MB EPS)

Figure S5 T cell subsets were purified from blood by conventional FACS markers. Sorting of naïve, effector memory (TEM), and central memory (TCM) CD4+ T cells from peripheral blood mononuclear cells. Numbers within plots indicate the fraction of cells selected. (a) Lymphocytes selected by forward/side scatter. (b) CD4+ cell selection. (c) CD4+ cells partitioned into CD45RO+ memory cells and CD45RA+ naïve cells. (d) CD45RO+ memory T cells partitioned into CD62L+CCR7+TCM cells and CD62- CCR7- TEM cells. (e) CD45RA+ naïve cells purified slightly further by selecting CD62L+CCR7+cells.
(0.68 MB EPS)

Figure S6 Matrix condition number guides the selection of the number of basis genes. Calculation of kappa, the matrix condition number, for various sizes of basis matrices. The basis size on the x-axis is the number of probesets selected from each statistical test for each constituent used in the basis matrix.
(0.49 MB EPS)

## References

[1] Rivero SJ, Diaz-Jouanen E, Alarcon-Segovia D (1978). Lymphopenia in systemic lupus erythematosus. Clinical, diagnostic, and prognostic significance.. Arthritis Rheum.

[2] Blanco P, Palucka AK, Gill M, Pascual V, Banchereau J (2001). Induction of dendritic cell differentiation by IFN-alpha in systemic lupus erythematosus.. Science.

[3] Gaipl US, Voll RE, Sheriff A, Franz S, Kalden JR (2005). Impaired clearance of dying cells in systemic lupus erythematosus.. Autoimmun Rev.

[4] Lub-de Hooge MN, de Vries EG, de Jong S, Bijl M (2005). Soluble TRAIL concentrations are raised in patients with systemic lupus erythematosus.. Ann Rheum Dis.

[5] Baechler EC, Batliwalla FM, Karypis G, Gaffney PM, Ortmann WA (2003). Interferon-inducible gene expression signature in peripheral blood cells of patients with severe lupus.. Proc Natl Acad Sci U S A.

[6] Bennett L, Palucka AK, Arce E, Cantrell V, Borvak J (2003). Interferon and granulopoiesis signatures in systemic lupus erythematosus blood.. J Exp Med.

[7] Sigurdsson S, Nordmark G, Goring HH, Lindroos K, Wiman AC (2005). Polymorphisms in the tyrosine kinase 2 and interferon regulatory factor 5 genes are associated with systemic lupus erythematosus.. Am J Hum Genet.

[8] Graham RR, Kozyrev SV, Baechler EC, Reddy MV, Plenge RM (2006). A common haplotype of interferon regulatory factor 5 (IRF5) regulates splicing and expression and is associated with increased risk of systemic lupus erythematosus.. Nat Genet.

[9] Remmers EF, Plenge RM, Lee AT, Graham RR, Hom G (2007). STAT4 and the risk of rheumatoid arthritis and systemic lupus erythematosus.. N Engl J Med.

[10] Speed T (2003). Statistical Analysis of Gene Expression Microarray Data.

[11] Subramanian A, Tamayo P, Mootha VK, Mukherjee S, Ebert BL (2005). Gene set enrichment analysis: a knowledge-based approach for interpreting genome-wide expression profiles.. Proc Natl Acad Sci U S A.

[12] Abbas AR, Baldwin D, Ma Y, Ouyang W, Gurney A (2005). Immune response in silico (IRIS): immune-specific genes identified from a compendium of microarray expression data.. Genes Immun.

[13] Lu P, Nakorchevskiy A, Marcotte EM (2003). Expression deconvolution: a reinterpretation of DNA microarray data reveals dynamic changes in cell populations.. Proc Natl Acad Sci U S A.

[14] Wang M, Master SR, Chodosh LA (2006). Computational expression deconvolution in a complex mammalian organ.. BMC Bioinformatics.

[15] Maas K, Chan S, Parker J, Slater A, Moore J (2002). Cutting edge: molecular portrait of human autoimmune disease.. J Immunol.

[16] Green MR, Kennell AS, Larche MJ, Seifert MH, Isenberg DA (2007). Natural killer T cells in families of patients with systemic lupus erythematosus: their possible role in regulation of IGG production.. Arthritis Rheum.

[17] Papadimitraki ED, Choulaki C, Koutala E, Bertsias G, Tsatsanis C (2006). Expansion of toll-like receptor 9-expressing B cells in active systemic lupus erythematosus: implications for the induction and maintenance of the autoimmune process.. Arthritis Rheum.

[18] Santini SM, Lapenta C, Logozzi M, Parlato S, Spada M (2000). Type I interferon as a powerful adjuvant for monocyte-derived dendritic cell development and activity in vitro and in Hu-PBL-SCID mice.. J Exp Med.

[19] Richez C, Yasuda K, Watkins AA, Akira S, Lafyatis R (2009). TLR4 ligands induce IFN-alpha production by mouse conventional dendritic cells and human monocytes after IFN-beta priming.. J Immunol.

[20] Helbig KJ, Lau DT, Semendric L, Harley HA, Beard MR (2005). Analysis of ISG expression in chronic hepatitis C identifies viperin as a potential antiviral effector.. Hepatology.

[21] Crow MK (2007). Type I interferon in systemic lupus erythematosus.. Curr Top Microbiol Immunol.

[22] Green MR, Kennell AS, Larche MJ, Seifert MH, Isenberg DA (2005). Natural killer cell activity in families of patients with systemic lupus erythematosus: demonstration of a killing defect in patients.. Clin Exp Immunol.

[23] Xu L, Zhang L, Yi Y, Kang HK, Datta SK (2004). Human lupus T cells resist inactivation and escape death by upregulating COX-2.. Nat Med.

[24] Hahn BH, Ebling F, Singh RR, Singh RP, Karpouzas G (2005). Cellular and molecular mechanisms of regulation of autoantibody production in lupus.. Ann N Y Acad Sci.

[25] Chan RW, Lai FM, Li EK, Tam LS, Chow KM (2006). Imbalance of Th1/Th2 transcription factors in patients with lupus nephritis.. Rheumatology (Oxford).

[26] Lit LC, Wong CK, Li EK, Tam LS, Lam CW (2007). Elevated gene expression of Th1/Th2 associated transcription factors is correlated with disease activity in patients with systemic lupus erythematosus.. J Rheumatol.

[27] Davis P, Percy JS, Russell AS (1977). Correlation between levels of DNA antibodies and clinical disease activity in SLE.. Ann Rheum Dis.

[28] Rastin M, Hatef MR, Tabasi N, Sheikh A, Morad Abbasi J (2007). Sex hormones and peripheral white blood cell subsets in systemic lupus erythematosus patients.. Iran J Immunol.

[29] Bonelli M, Savitskaya A, Steiner CW, Rath E, Smolen JS (2009). Phenotypic and functional analysis of CD4+CD25−Foxp3+ T cells in patients with systemic lupus erythematosus.. J Immunol.

[30] Zimmer J, Andres E, Hentges F (2008). NK cells and Treg cells: a fascinating dance cheek to cheek.. Eur J Immunol.

[31] Pisetsky DS (2008). The role of innate immunity in the induction of autoimmunity.. Autoimmun Rev.

[32] Lahdesmaki H, Shmulevich L, Dunmire V, Yli-Harja O, Zhang W (2005). In silico microdissection of microarray data from heterogeneous cell populations.. BMC Bioinformatics.

[33] Bar-Joseph Z, Farkash S, Gifford DK, Simon I, Rosenfeld R (2004). Deconvolving cell cycle expression data with complementary information.. Bioinformatics.

[34] Buess M, Nuyten DS, Hastie T, Nielsen T, Pesich R (2007). Characterization of heterotypic interaction effects in vitro to deconvolute global gene expression profiles in cancer.. Genome Biol.

[35] Team RDC (2008). R: A Language and Environment for Statistical Computing.

