# Supplementary material for: Deconvolution of Blood Microarray Data Identifies Cellular Activation Patterns in Systemic Lupus Erythematosus
Source: PLoS One. 2009 Jul 1;4(7):e6098. doi: 10.1371/journal.pone.0006098 (PMC2699551; doi:10.1371/journal.pone.0006098)
Supplement: Table S1 — Mean expression data for the pure cell types and the probesets used as the basis matrix for performing expression deconvolution on whole blood samples from the main clinical test cohort or on the validation set of purified leukocytes. (0.11 MB PDF) [file pone.0006098.s001.pdf]

Probeset	Entrez	Symbol	Name	Th	Th act	Tc	Tc act	B	B act	B aIgM	Mem IgG	Mem IgM	PC	NK	NK act	mono	mono act	DC	DC act	neutro
242904_x_at				5292.9	104.6	191.29	41.553	671.33	125.9	78.485	1355.6	2167.6	575.7	234.22	193.38	60.43	94.563	44.379	42.333	528.59
232165_at				605.1	57.129	19.282	23.457	19.076	22.296	18.152	51.534	45.244	24.699	22.594	25.374	15.907	23.304	17.794	14.671	49.81
201369_s_at	678	ZFP36L2	zinc finger protein 36, C3	5213.9	625.69	860.42	267.09	1040.9	563.83	248.18	422.51	430.73	170.09	944.54	829.88	947.23	303.25	768	224.49	685.29
233127_at				1710.6	222.72	147.83	79.293	319.04	236.36	156.19	134.89	262.19	128.64	118.91	74.872	49.12	36.745	26.144	19.642	24.981
204621_s_at	4929	NR4A2	nuclear receptor subfami	1407.8	247.46	19.61	143.09	50.766	112.38	322.4	202.34	365.71	42.58	16.994	108.1	62.992	220.11	49.329	100.96	106.7
202861_at	5187	PER1	period homolog 1 (Droso	1079.4	104.49	189.22	154.5	57.445	81.581	162.01	48.368	116.49	31.034	102.67	116.49	189.11	128.72	103	82.624	72.219
222862_s_at	26289	AK5	adenylate kinase 5	1379.6	330.29	203.27	52.567	50.005	48.406	38.379	69.45	65.663	55.256	199.32	175.49	27.348	33.242	26.782	42.736	114.44
235213_at				9321.4	1863.7	1799	613.54	1615.5	2073.6	2786.8	231.11	151.04	294.67	2300	1248	134.46	135.6	290.04	94.781	610.74
235739_at	4929	NR4A2	nuclear receptor subfami	6951	389.67	369.9	408.86	1149.1	388.4	630.63	1875.9	3071.5	448.05	192.38	340.33	209.66	263.31	151.63	182.99	1779.9
231798_at	9241	NOG	noggin	1205.8	216.29	175.73	61.715	163.99	62.575	71.163	153.18	210.55	226.19	108.3	65.022	67.638	107.4	66.928	68.087	245.57
200664_s_at	3337	DJB1	DnaJ (Hsp40) homolog, s	4361.3	980.92	842.13	919.73	580.86	779.84	629.17	342.02	318.99	888.9	913.3	898.93	1674.3	1546.3	1153.8	890.5	815.13
240452_at				3343.6	1173.4	661.92	751.8	737.37	768.24	920.58	513.34	503.68	565.05	460.41	371.28	148.31	138.81	121.14	117.47	234.13
236907_at				14624	2674.3	1387.4	3255.3	5202	1429.3	2599.7	3060.4	3890.8	1513.1	773.73	774.08	460.05	327.9	234.19	363.43	1545.7
204622_x_at	4929	NR4A2	nuclear receptor subfami	1142.3	259.03	75.925	204.75	125.36	209.68	312.83	502.61	820.81	97.28	167.22	211.2	153.38	325.82	153.57	127.94	217.77
227307_at				1948.2	400.21	234	129.54	353.61	210.34	149.64	323.63	239.4	712.5	93.089	110.13	83.97	121.68	134.12	124.52	230.54
227641_at	146330	FBXL16	F-box and leucine-rich re	3811.3	1576.5	1403.5	697.89	732.34	672.19	494.3	833.77	550.58	1161	311.67	323.29	224.19	245.56	351.13	401.22	1170.8
201367_s_at	678	ZFP36L2	zinc finger protein 36, C3	3793.8	620.67	771.55	219.34	1023.5	486.97	268.09	366.91	415.21	172.63	893.78	701.58	1109.2	276.4	869.58	278.41	706.3
234408_at	112744	IL17F	interleukin 17F	75.661	9648.6	96.514	126.73	146.75	99.437	125.95	181.58	145.47	169.89	132.08	57.788	66.779	62.439	48.914	67.21	192.07
216876_s_at	3605	IL17A	interleukin 17A	7.194	809.34	3.396	10.259	7.24	8.1	3.219	24.895	36.767	5.585	6.173	21.649	12.938	15.326	13.615	19.02	18.389
234362_s_at	1493	CTLA4	cytotoxic T-lymphocyte-a	1152.6	5171.8	644.65	1132.7	400.95	331.91	319.33	599.21	486.96	540.88	280.2	272.35	127.25	202.8	219.86	213.03	546.89
221331_x_at	1493	CTLA4	cytotoxic T-lymphocyte-a	456.17	1826.6	170.64	539.95	77.447	57.268	68.635	58.954	76.762	89.278	105.53	117.46	74.196	74.198	166.69	144.29	185.74
211856_x_at	940	CD28	CD28 molecule	484.12	1102.4	350.46	347.35	148.4	101.36	39.628	175.46	291.21	134.16	54.782	99.024	69.36	108.31	103.85	99.261	313.67
219179_at	51339	DACT1	dapper, antagonist of bet	276.97	664.71	99.844	95.204	207.63	228.89	116.55	212.18	274.44	115.61	56.953	114.38	164.18	108.19	238.82	129.36	226.99
221165_s_at	50616	IL22	interleukin 22	195.27	1621.9	140.2	272.41	182.77	177	131.76	305.89	259.44	170.05	130.08	140.77	160.41	208.94	195.76	176.95	458.47
235150_at				848.53	1665.6	388.3	162.9	305.6	519.98	590.79	135.39	151.2	120.16	479.75	251.81	70.653	94.283	209.79	790.54	79.465
235616_at	128553	TSHZ2	teashirt zinc finger home	669.63	1663.3	288.41	312.36	171.51	286.96	117.25	355.25	272.75	201.94	169.31	130.05	81.735	121.52	97.362	109.71	253.85
227262_at	145864	HAPLN3	hyaluronan and proteogl	1946.6	8728.1	1101.6	2305.8	594.52	1180.4	388.18	558.27	536.28	992.28	563.5	2809.2	254.6	430.41	267.09	1769.6	644.6
226913_s_at	30812	SOX8	SRY (sex determining reg	298.04	1025	19.781	21.533	30.567	26.109	23.557	28.412	27.116	41.972	39.268	45.119	15.518	25.178	50.898	22.052	25.256
211861_x_at	940	CD28	CD28 molecule	424.1	927.04	284.99	249.87	32.017	33.209	17.453	61.339	66.092	45.075	65.611	35.191	29.141	49.231	19.951	27.802	37.221
231794_at	1493	CTLA4	cytotoxic T-lymphocyte-a	829.63	2074.9	360.49	772.02	172.99	225.83	82.15	277.45	353.17	308.49	124.66	116.43	92.171	92.422	121.75	109.83	501.04
216901_s_at	10320	IKZF1	IKAROS family zinc finge	585.01	1251.5	289.46	343.31	289.61	355.95	186.75	251.73	257.34	202.66	571.49	213.94	254.88	233.08	271.55	439.89	210.19
220892_s_at	29968	PSAT1	phosphoserine aminotrar	346.27	2214.3	394.62	805.9	320.85	337.81	736.12	429.65	309.66	1025.6	199.65	207.83	202.32	287.5	581.74	510.86	477.33
208193_at	3578	IL9	interleukin 9	97.274	1343.5	49.426	410.4	83.163	57.007	22.289	145.32	101.28	54.427	95.923	63.72	52.85	60.811	68.764	52.046	88.46
243940_at	128553	TSHZ2	teashirt zinc finger home	305.87	591.41	112.12	137.34	154.73	110.94	63.719	214.32	219.95	234.12	102.72	72.86	28.29	63.443	42.089	40.373	163.89
215332_s_at	926	CD8B	CD8b molecule	41.855	32.983	1489.1	122.68	37.742	27.152	8.683	93.873	74.936	50.758	66.688	29.283	42.361	57.728	46.062	30.886	58.733
209840_s_at	54674	LRRN3	leucine rich repeat neuro	735.62	503.37	6792.8	1198.6	62.117	40.136	28.776	157.23	118.26	33.943	46.41	34.332	99.24	152.85	121.16	87.014	107.64
205758_at	925	CD8A	CD8a molecule	310.06	405.99	9957.8	3315.8	611.63	460.91	106.98	363.04	243.73	125.54	1747.3	1021.5	176.49	358.05	191.57	212.95	659.97
207979_s_at	926	CD8B	CD8b molecule	138.63	177.5	5811.1	2622.3	115.71	130.58	54.383	281.08	335.21	118.48	239.29	169.83	138.51	232.77	128.76	116.27	422.64
209841_s_at	54674	LRRN3	leucine rich repeat neuro	706.71	581.81	8644.5	2115.9	159.72	143.34	90.669	332.35	344.48	173.99	111.6	105.81	175.1	267.95	204.23	201.49	495.58
227915_at	51676	ASB2	ankyrin repeat and SOCS	79.337	112.51	2639.9	927.9	38.727	46.681	31.775	53.498	43.509	219.16	31.89	23.954	18.171	22.51	23.759	68.321	32.828
207232_s_at	9666	DZIP3	DAZ interacting protein 3	63.449	15.003	194.1	12.941	59.71	26.541	25.563	37.349	36.696	17.2	35.794	21.865	21.678	28.811	39.256	44.789	30.134
234427_at				246.02	176.44	613.76	234.81	24.22	30.823	24.564	39.521	42.166	31.209	23.72	13.456	19.854	21.666	14.435	20.995	53.299
226423_at	85315	PAQR8	progestin and adipoQ rec	786.88	1044.9	2261.4	883.48	460.26	520.17	258.38	424.19	344.92	557.11	945.21	671.94	472.6	224.57	882.61	233.68	556.69
208406_s_at				102.7	186.89	569.46	181.94	24.59	16.219	12.373	19.32	20.428	13.39	189.79	247.42	18.973	24.599	22.105	22.97	27.632
217119_s_at	2833	CXCR3	chemokine (C-X-C motif)	232.2	123.87	1592.3	282.94	73.401	52.68	25.821	101.19	79.587	242.77	602.71	345.71	49.709	78.357	64.907	28.424	62.056
242628_at				237.41	181.43	750.95	276.46	270.68	110.13	115.56	162.12	76.456	206.92	278.26	253.8	41.45	39.372	63.174	30.798	241.79
201414_s_at	4676	P1L4	nucleosome assembly pr	1095.9	1054.6	1961.3	1146	851.07	858.5	642.55	569.35	587.16	796.58	1028.9	774.41	1198.6	1045.5	896.23	1096.3	451.85
227552_at	1731	38230	septin 1	1853.2	1078	3456.8	1540.5	1236.1	1119.3	944.65	371.66	442.15	1079.7	1159.2	975.22	21.243	61.854	26.316	69.847	148.47
241871_at	814	CAMK4	calcium/calmodulin-depe	1278.2	978.5	2274.6	1265.9	238.29	296.08	68.922	188	231.35	301.28	139.73	101.14	25.46	75.621	50.817	142.6	204.37
219025_at	57124	CD248	CD248 molecule, endosia	366.65	216.83	1028.2	288.42	329.16	282.39	195.08	324.75	255.77	252.92	213.8	213.93	317.03	255.8	371.85	289.84	453.31
234398_at				116.38	108.86	526.68	257.03	74.15	61.25	39.666	104.62	117.43	122.23	49.748	27.014	31.759	55.044	32.022	36.939	217.53
243110_x_at	283869	NPW	neuropeptide W	5.177	5.275	4.551	539.25	6.061	5.692	446.58	11.427	16.884	12.242	4.098	4.461	7.843	11.752	8.392	9.127	7.039
227617_at	199953	RP13-15M	hypothetical protein LOC	192.86	235.07	220.53	3249.7	173.54	170.17	95.56	141.98	147.22	290.42	134.75	135.83	100.88	112.42	107.51	102.01	213.66
235238_at	399694	SHC4	SHC (Src homology 2 do	59.433	60.769	40.265	570.24	33.133	62.257	34.752	92.555	87.079	47.668	21.764	31.468	18.077	29.599	26.114	25.334	60.573
205266_at	3976	LIF	leukemia inhibitory facto	31.201	1084.5	36.214	5823.8	25.66	21.031	19.177	49.072	46.371	25.724	20.468	84.797	104.49	625.31	55.962	48.533	66.225
209000_s_at	23176		septin 8	11.013	20.212	16.933	126.6	10.758	11.885	13.505	25.628	24.617	18.216	13.231	10.118	20.444	23.372	23.637	21.66	24.944
203967_at	990	CDC6	cell division cycle 6 homo	93.088	194.93	220.38	1223.1	107.62	112.04	83.857	174.97	175.32	310.89	80.572	41.998	52.73	66.45	58.133	41.306	272.02
223490_s_at	51010	EXOSC3	exosome component 3	118.67	789.6	738.75	2638.3	174.02	567.47	822.37	48.067	79.427	299.82	499.31	691.75	285.35	269.96	183.8	226.82	93.13
224851_at	1021	CDK6	cyclin-dependent kinase	3018.2	4621.4	3289.6	19509	2481.6	2276.6	1490.8	493.55	258.8	3295	1248.6	830.99	475.77	235.09	1131.8	1444.6	318.97

218529_at	51293	CD320	CD320 molecule	144.21	273.65	232.18	1229.6	68.268	144.65	283.68	59.468	53.224	315.2	113.03	29.97	59.036	145.06	75.737	63.493	39.181
202613_at	1503	CTPS	CTP synthase	233.48	828.46	608.83	2722.2	268.05	532.91	994.27	373.66	397.97	386.96	223.21	303.98	430.55	239.9	573.47	332.52	293.59
224523_s_at	84319	C3orf26	chromosome 3 open rea	844.34	1873.3	1306.2	6709.1	935.86	1139.8	3038.4	411.69	346.84	1622.2	427.68	588.48	366.89	183.34	348.61	236.71	368.07
206653_at	10622	POLR3G	polymerase (R) III (DNA	111.28	129.25	80.188	499.74	110.15	90.279	191.06	105.25	95.599	122.2	65.923	66.872	62.421	96.927	95.214	73.507	98.346
228671_at	199953	RP13-15M	hypothetical protein LOC	169.99	261.11	249.09	1122.7	140.65	160.81	171.54	123.62	129.9	114.47	115.85	116.68	56.981	51.74	109.25	33.679	89.197
224848_at	1021	CDK6	cyclin-dependent kinase	1750.7	2549.3	1791.3	9645	1502.4	1408.9	897.3	752.27	632.6	2131.3	830.37	552.98	397.79	296.18	766.85	720.85	518.97
203270_at	1841	DTYMK	deoxythymidylate kinase	108.09	195.01	278.64	1007.2	163.8	160.07	273.64	133.27	153.84	292.14	178.37	112.05	210.83	157.39	223.51	179.2	45.123
205164_at	23464	GCAT	glycine C-acetyltransfera	20.973	14.22	28.833	191.85	16.681	11.739	14.28	22.541	21.448	16.567	17.535	9.065	24.936	33.227	23.741	24.881	37.288
205133_s_at	3336	HSPE1	heat shock 10kDa protei	844.06	2476.6	1665.5	7532.5	669.89	1505.3	2903.3	846.93	783.39	1572.6	605.63	767.69	1753.7	1231	2327	1763.6	188.67
236820_at				59.386	43.757	62.947	48.205	1755	162.4	482.29	413.11	121.42	128.14	60.648	39.065	34.083	55.897	28.843	37.901	114.38
215925_s_at	971	CD72	CD72 molecule	58.037	36.506	120.52	81.1	2131.4	429.63	938.19	245.96	573.81	17.596	140.01	70.636	86.43	25.64	98.177	38.692	20.212
204581_at	933	CD22	CD22 molecule	15.819	9.314	10.67	9.456	4220.4	899.99	1028.4	411.99	546.56	11.084	17.766	12.93	105.23	68.891	59.415	28.518	31.877
216766_at				163.92	86.114	48.873	16.798	1334.1	244.78	244.54	171.12	212.56	22.94	70.721	45.52	19.657	34.482	25.068	27.183	27.135
210432_s_at	6328	SCN3A	sodium channel, voltage-	9.889	14.549	3.845	5.263	722.63	159.39	75.757	124.23	81.037	22.318	9.035	16.917	20.771	16.759	15.008	14.52	30.192
224329_s_at	84518	CNFN	cornifelin	17.525	20.474	15.638	15.914	223.47	42.35	25.314	47.823	161.18	31.814	13.975	11.258	14.451	23.411	16.705	19.943	27.996
39318_at	8115	TCL1A	T-cell leukemia/lymphor	413.37	244.87	53.831	83.168	11712	2673.8	2889.4	498.98	831.45	293.26	180.04	170.35	119.83	297.68	185.86	228.53	348.28
220068_at	29802	VPREB3	pre-B lymphocyte gene 3	155.11	68.624	42.995	57.886	2850.9	755.75	264.61	1466.2	1287.6	178.78	61.713	94.775	90.293	96.477	78.715	97.26	123.4
235460_at	79856	SNX22	sorting nexin 22	47.638	48.233	40.53	35.855	607.56	124.46	101.79	348.5	401.1	104.37	44.03	51.925	27.59	49.441	36.764	35.319	111.72
230385_at				81.96	51.288	73.467	72.773	345.25	86.994	110.34	75.808	75.208	61.51	39.875	54.774	28.204	37.371	32.484	30.718	88.844
209995_s_at	8115	TCL1A	T-cell leukemia/lymphor	641.02	399.85	114.45	76.966	13310	3401.9	2895.9	575.35	902.55	275.69	286.46	251.84	247	312.07	191.15	185.85	392.58
239655_at				915.56	281.78	499.02	263.74	3325	798.08	831.82	778	802.7	432.14	407.7	331.35	113.97	184.05	84.152	122.42	456.53
239292_at				221.07	128.11	19.172	32.89	1833	502.73	422.8	785.39	690.67	272.74	31.909	47.045	11.475	22.142	16.219	13.423	120.6
230245_s_at	283663	LOC28366	hypothetical protein LOC	726.74	166.83	123	126.47	23022	6478.5	4462	6503.1	7265.8	226.35	265.95	221.32	914.55	1401.3	239.32	423.65	219.41
230648_at	283663	LOC28366	hypothetical protein LOC	692.64	453.05	520.19	392.15	13389	4016.6	2195.3	3613.9	4563.9	556.32	476.24	428.56	669.75	1095.6	309.15	406.34	897.64
38521_at	933	CD22	CD22 molecule	390.89	254.78	175.49	153.74	5953.5	1829.8	1631.8	1793.3	2011	266.97	243.9	251.42	697.13	618.3	649.58	454.74	714.06
218949_s_at	55278	QRSL1	glutaminyl-tR synthase (	387.5	465.19	526.61	783.83	2643	875.1	727.76	1464.3	1714.3	621.96	354.9	363.68	464.82	337.89	791.38	630.87	176.49
234284_at	94235	GNG8	guanine nucleotide bindi	53.579	611.56	23.299	184.36	51.681	8705.2	681.42	43.5	64.063	57.214	16.072	16.477	17.853	61.13	14.916	18.059	41.406
212094_at	23089	PEG10	paternally expressed 10	46.498	31.167	66.482	18.336	253.9	2036.6	121.93	81.024	103.77	73.413	54.027	25.162	34.173	64.445	37.859	21.48	90.119
227193_at				743.61	680.67	534.2	492.15	1318.3	7943.9	326.7	1065.5	710.81	847.84	805.39	1089.5	85.611	279.09	740.4	422.82	497.14
223241_at	29886	SNX8	sorting nexin 8	72.247	269.15	243.03	479.83	451.44	3818.5	2789.4	150.52	188.22	240.7	122.51	111.59	513.67	723.39	598.57	235.17	29.861
242468_at				121.46	90.123	93.263	76.315	493.97	2956.6	774.48	232.44	167.55	182.61	91.025	91.24	45.43	58.428	42.262	45.095	185.27
227066_at	148932	MOBKL2C	MOB1, Mps One Binder k	879.19	2018.4	1343.5	1029.6	825.81	13894	717.4	604.51	685.16	895.23	1533.5	3005.6	1422	1492.7	1051.1	937.14	980.55
219517_at	80237	ELL3	elongation factor R polyn	156.69	158.02	95.262	104.13	503.88	2672.8	627.44	196.76	187.92	102	132.33	109.34	241.61	198.86	252.73	569.53	256.98
226274_at	1184	CLCN5	chloride channel 5 (neph	757.15	676.29	495.2	584.13	623.52	4105.2	524.36	1044.6	1151.2	764.8	628.32	554.57	767.28	555.77	891.8	452.43	1058.2
235142_at	653121	ZBTB8	zinc finger and BTB doma	163.85	107.16	57.552	97.531	219.78	2444	154.17	222.47	278.34	440.52	133.21	138.24	97.11	73.722	383.62	117.5	352.18
228231_at				53.024	126.27	68.75	269.56	451.93	3470.4	881.09	127.17	231.04	56.611	98.693	36.689	294.02	279.9	911.37	208.28	38.002
232127_at	1184	CLCN5	chloride channel 5 (neph	155.94	162.89	109.18	181.57	159.9	1331.7	179.77	216.7	184.82	145.23	123.85	106.44	244.96	215.04	243.33	146.17	260.36
227006_at	94274	PPP1R14A	protein phosphatase 1, r	30.346	9.07	9.096	9.518	392.81	2681.6	109.95	196.29	198.03	100.7	11.632	11.142	9.568	26.704	96.818	610.68	22.289
219518_s_at	80237	ELL3	elongation factor R polyn	159.7	130.7	92.114	118.93	420.44	1706	524.31	229.49	264.89	170.12	146.24	169.55	197.8	185.91	202.84	505.74	244.67
209101_at	1490	CTGF	connective tissue growth	22.249	22.905	6.739	3.515	43.341	265.32	34.172	29.803	13.406	12.285	15.781	21.534	11.813	18.578	17.114	41.291	35.862
243052_at	148932	MOBKL2C	MOB1, Mps One Binder k	588.25	1078.3	640.07	622.65	514.18	4626	496.97	611.27	530.52	453.65	829.5	1255.6	710.25	607.66	533.21	447.56	630.08
235278_at	140733	C20orf133	MACRO domain containir	210.22	110.16	58.043	68.735	3510.6	11697	5016.8	124.2	181.28	644.92	79.989	66.195	42.426	76.844	58.566	40.426	140.71
241483_at				177.14	95.259	75.054	51.036	183.49	657.76	44.223	102.93	129.5	188.89	144.92	97.92	42.783	47.617	43.627	37.469	164
223565_at	51237		hypothetical protein MGC	768.21	1072	205.82	123.61	2046.6	1716.7	785.93	740.4	838.1	37183	52.632	104.09	19.182	52.139	32.979	18.784	67.427
211835_at	652070		single-chain Fv fragment	17.024	12.551	9.378	10.146	26.928	11.946	14.494	23.924	21.578	677.99	15.836	17.732	23.55	32.221	33.423	26.31	32.073
233969_at				27.368	28.913	16.45	26.404	135.1	40.005	74.637	57.895	39.922	5202.1	22.518	18.29	18.087	22.137	20.973	43.664	43.749
234792_x_at				476.89	528.5	108.54	42.032	957.48	592.6	712.36	296.25	472.3	32592	92.062	77.439	21.719	38.212	25.846	35.352	64.939
221004_s_at	81618	ITM2C	integral membrane prote	585.42	318.44	534.58	194.83	1031.8	738.28	247.2	577.24	779.46	11437	542.99	323.12	33.527	83.342	50.067	43.392	129.57
221286_s_at	51237		hypothetical protein MGC	506.82	307.29	21.088	21.828	1374.2	1319.5	490.69	260.48	239.29	17479	16.949	17.981	29.661	37.977	33.723	31.289	40.291
206641_at	608	TNFRSF17	tumor necrosis factor rec	481.56	79.328	7.069	4.69	1187.8	365.71	263.56	626.98	442.39	14375	25.957	9.114	21.037	21.426	26.508	35.455	32.888
211641_x_at				669.73	445.68	99.639	82.5	1858	900.13	604.22	1247.4	2371.6	22774	146.08	117.63	154.76	164.45	135.49	142.59	353.57
224342_x_at				1159.5	1240.9	243.81	144.13	4950.9	3547.4	1665.6	1460.3	1353.7	44647	76.908	111.4	74.646	119.1	83.672	98.779	225.79
216412_x_at				132.76	183.85	15.044	25.193	757.95	436.2	527.22	512.07	467.82	6583.2	44.886	41.648	29.585	73.693	31.832	47.005	75.732
219118_at	51303	FKBP11	FK506 binding protein 11	1010.8	1771.9	1287	1715.5	833.49	736.14	618.66	596.84	431.99	13740	951.93	1748.7	153.69	225.52	152.68	102.36	170.24
234419_x_at				107.22	119.57	35.991	31.263	429.37	167.16	108.45	65.809	55.434	4308.9	28.589	30.469	25.767	36.928	23.977	25.044	43.659
217217_at				18.285	15.852	9.172	7.818	47.27	47.245	16.959	37.188	22.782	555.37	14.155	11.369	21.694	31.114	22.223	19.143	23.746
217179_x_at				589.65	458.26	148.22	85.462	3392.6	2245	842.29	1265.4	1225.6	26624	43.581	136.18	116.63	117.3	107.23	160.4	243.01
219003_s_at	79694	MANEA	mannosidase, endo-alpha	87.389	192.19	113.36	190.16	109.12	128.83	61.721	57.43	51.351	1510.6	85.751	124.28	111.99	82.277	196.15	182.86	5.879
217258_x_at				316.7	249.63	21.981	8.441	1613	1120.6	591.59	697.15	670.13	13019	31.014	20.293	21.717	30.131	21.158	20.484	70.638
212097_at	857	CAV1	caveolin 1, caveolae prot	45.356	22.639	15.996	72.569	96.76	64.758	65.577	46.166	24.508	1889.2	14.734	23.886	23.042	136.13	51.28	156.41	46.812

221383_at	10316	NMUR1	neuromedin U receptor 1	16.485	10.581	10.655	9.429	23.474	16.968	11.241	28.366	24.025	21.331	172.26	28.295	24.576	43.296	32.587	28.674	25.14
228948_at	2043	EPHA4	EPH receptor A4	232.57	88.394	232.39	42.03	171.22	92.377	36.349	140.79	81.496	54.073	2761.4	735.14	25.869	40.016	27.074	27.474	82.083
212062_at	10079	ATP9A	ATPase, Class II, type 9A	11.562	14.731	6.817	9.387	12.617	10.289	11.696	19.097	25.438	10.548	948.85	203.48	40.959	55.259	160.09	41.003	27.762
212070_at	9289	GPR56	G protein-coupled recept	450.23	46.85	680.21	378.36	273.85	146.6	17.949	47.686	42.818	31.702	5945.4	1929.6	50.639	140.34	46.329	72.247	180.88
206114_at	2043	EPHA4	EPH receptor A4	304.21	44.721	162.55	12.343	158.35	137.15	20.26	166.81	42.352	38.186	1388.8	396.3	25.82	30.08	30.872	25.258	53.499
217889_s_at	79901	CYBRD1	cytochrome b reductase	7.287	5.64	5.593	5.551	9.848	8.445	7.804	19.536	17.719	8.519	121.89	7.789	21.734	21.921	33.831	21.927	19.441
216050_at				56.285	18.537	102.77	15.889	50.579	25.757	25.26	112.58	61.378	25.283	535.44	147.87	18.017	23.31	13.838	26.439	28.242
228108_at				264.55	96.483	257.25	150.53	209.8	240.56	152.7	259.38	192.84	210.88	1220.4	304.67	157.85	96.387	413.32	116.43	274.48
223126_s_at	81563	C1orf21	chromosome 1 open read	150.22	48.558	482.41	176.14	151.32	97.005	29.468	93.735	57.454	63.917	2150.6	903.11	84.932	390.11	31.397	23.844	159.08
204457_s_at	2619	GAS1	growth arrest-specific 1	12.055	10.149	9.647	9.038	20.012	12.016	8.361	24.734	25.129	12.794	178.7	35.304	17.643	34.257	42.159	26.652	44.204
226247_at	59338	PLEKHA1	pleckstrin homology dom	2609.8	1355.1	1304.4	665.37	1768.9	1304.9	762.61	848.99	955.81	356.19	7208.2	2925.4	617.2	652.31	909.71	1482.1	442.03
229374_at	2043	EPHA4	EPH receptor A4	900.99	422.48	455.53	254.47	417.9	429.13	273.55	717.74	602.07	596.28	2364.5	967.61	145.92	239.82	138.98	172.69	440
211209_x_at	4068	SH2D1A	SH2 domain protein 1A,	390.85	464.3	737.65	455.64	77.492	72.491	74.097	47.594	46.695	20.921	1693	746.65	51.221	51.22	39.309	41.259	66.209
227449_at	2043	EPHA4	EPH receptor A4	634.35	162.79	615.75	128.7	195.17	190.16	66.138	395.22	109.34	155.65	3974.9	1121.7	19.352	28.697	24.173	24.334	157.95
206355_at	2774	GL	guanine nucleotide bindi	85.932	63.9	41.075	28.466	74.428	43.235	33.021	90.869	85.197	79.187	193.61	90.085	39.564	54.834	62.992	63.343	84.722
243951_at				281.26	147.7	424.32	150.74	410.31	274.03	186.01	228.02	225.67	224.27	1488.5	773.91	78.088	55.655	48.71	46.583	298.63
226279_at	11098	PRSS23	protease, serine, 23	337.78	158.02	147.29	189.44	245.58	359.39	132.8	330.03	377.04	351.5	2740.9	775.99	92.684	108.22	107.02	101.46	407.85
229973_at	127254	C1orf173	chromosome 1 open read	53.192	88.232	19.387	54.307	43.526	75.933	42.083	112.26	106.76	33.029	96.537	5221.1	20.035	16.235	31.973	19.899	76.112
230926_s_at	57489	ODF2L	outer dense fiber of sper	402.52	524.06	396.31	137.76	355.88	155.38	159.46	183.49	113.26	312.66	469.39	2852.3	29.954	88.016	51.95	64.323	111.68
228577_x_at	57489	ODF2L	outer dense fiber of sper	361.13	401.41	336.7	142.21	219.51	187.77	125.38	240.69	116.48	274.85	401.76	2054.4	33.763	61.725	39.819	107.49	86.458
229893_at	257019	FRMD3	FERM domain containing	304.9	315.32	121.07	156.3	292.62	214.85	117.87	312.93	474.35	213.8	284.1	1797.8	111.72	76.125	133.01	202.21	294.68
203571_s_at	10974	C10orf116	chromosome 10 open re	13.1	8.346	7.777	5.934	22.182	27.798	11.084	22.65	20.283	15.804	11.696	225.5	19.308	40.143	28.946	25.319	23.581
210218_s_at	6672	SP100	SP100 nuclear antigen	301.3	711.82	491.39	248.45	889.83	574.84	396.32	225.06	197.43	120.03	990.02	3884	572.42	755.13	276.02	661.01	630.04
219684_at	64108	RTP4	receptor (chemosensory)	156.03	1646.5	521.18	124.92	92.382	560.78	58.119	59.991	60.383	224.17	836.07	6115.6	346.26	438.55	577.13	1663.7	64.385
224973_at	55603	FAM46A	family with sequence sim	209.46	254.63	56.082	30.957	262.27	338.22	140.46	70.072	70.491	96.183	750.31	2775.4	406.81	129.88	401.53	597.47	211.98
228531_at	54809	SAMD9	sterile alpha motif domai	878.92	5053.9	3029.7	540.35	1463	1883.7	500	389.29	544.59	1078.5	4316.6	19829	471.56	584.64	274.6	2650.4	1593.1
230645_at	257019	FRMD3	FERM domain containing	290.98	504.45	214.86	121.57	355.76	267.45	226.52	846.65	811.22	387.72	223.91	3689.8	94.875	77.308	140.16	266.24	748.24
226103_at	91624	NEXN	nexilin (F actin binding p	67.656	627	67.589	118.05	87.618	101.62	150.77	82.89	38.41	30.593	115.11	2299.9	17.823	28.107	16.964	51.955	64.69
236156_at	3988	LIPA	lipase A, lysosomal acid,	34.715	451.51	34.551	44.167	32.135	29.07	32.918	52.246	56.176	76.157	21.641	2082.2	39.556	27.231	22.498	96.451	70.775
210705_s_at	85363	TRIM5	tripartite motif-containin	177.65	345.55	336.22	108.71	330.94	318.82	274.03	143.37	183.16	317.66	431.34	1212.8	328.47	236.23	326.44	417.5	213.38
218501_at	50650	ARHGEF3	Rho guanine nucleotide e	1839.8	2785.7	3143.2	928.31	802.1	999.06	266.06	374.46	472.91	440.33	4649.5	10693	808.73	521.37	1982.5	1260	265.32
228675_at				321.04	662.29	499.97	368.98	350.57	250.69	163.87	200.3	148.86	637.26	430.83	2257.5	109.55	72.205	85.077	413.35	267.85
236191_at				190.68	1376.2	626.48	604.37	248.31	303.43	158.5	49.547	60.247	882.36	259.52	4757.8	25.85	56.1	24.379	190.18	128.97
223434_at	2635	GBP3	guanylate binding protein	704.07	2327.8	2251.5	1177	507.74	597.15	260.27	260.49	282.58	680.29	3614.4	9689.2	235.89	408.95	477.37	1973.1	442.26
201185_at	5654	HTRA1	HtrA serine peptidase 1	298.66	211.29	129.67	158.13	244.18	325.94	217.11	254.78	217.39	378.02	182.02	100.41	4524.5	721.17	344.37	393.46	408.27
211165_x_at	2048	EPHB2	EPH receptor B2	18.128	14.747	13.441	10.721	22.435	19.212	13.813	41.378	38.509	19.184	36.082	33.919	750	37.251	66.137	111.51	40.488
211913_s_at	10461	MERTK	c-mer proto-oncogene ty	170.5	109.41	114.19	123.07	217.36	151.04	110.88	131.29	147.68	167.79	155.64	143.83	2057.5	444.14	347.38	196.23	221.36
208771_s_at	4048	LTA4H	leukotriene A4 hydrolase	2762.8	3065.3	1893.5	2088.7	5716.5	5135.1	4152	2095.9	2279.2	3178.3	2278	832.7	21704	2697.1	4330.1	1290.8	1902.3
209589_s_at	2048	EPHB2	EPH receptor B2	30.863	44.763	17.499	32.405	59.756	19.794	14.376	68.248	49.623	86.171	54.592	51.961	1348.1	30.996	332.32	326.9	110.81
206028_s_at	10461	MERTK	c-mer proto-oncogene ty	190.69	107.8	58.619	53.798	117.56	104.65	56.653	236.55	207.85	107.1	126.88	50.745	3131.1	768.85	422.35	132.67	247.23
227823_at	340526	RGAG4	retrotransposon gag dom	78.208	111.4	61.529	63.163	103.33	94.134	75.628	96.029	94.257	95.477	112.3	123.43	933.54	233.76	297.81	126.5	147.74
235751_s_at	284013	VMO1	vitelline membrane outer	34.258	39.726	11.115	11.268	16.153	14.451	19.847	23.683	20.328	23.967	362.26	11.41	2665.1	396.29	97.002	261.12	21.038
236646_at	120939	C12orf59	chromosome 12 open re	47.859	43.294	34.943	30.558	36.092	85.694	45.534	69.956	54.285	134.15	72.249	101.61	821.29	191.39	106.19	115.88	143.71
218832_x_at	408	ARRB1	arrestin, beta 1	34.25	29.224	38.111	72.62	31.272	19.392	15.134	37.238	34.008	20.44	144	87.986	591.46	169.9	179.32	77.117	79.13
209588_at	2048	EPHB2	EPH receptor B2	197.37	142.57	136.13	141.82	204.37	159.93	109.4	240.71	226.39	202.71	117.7	98.776	1139.8	190.47	393.61	362.3	378.24
202838_at	2517	FUCA1	fucosidase, alpha-L- 1, ti	242.55	302.08	495.6	216.27	440.56	450.22	469.94	416.64	325.97	713.21	2269	1457.2	23621	1428.6	7804.2	2832.3	348.69
208862_s_at	51075	TXNDC14	thioredoxin domain conta	170.59	108.76	126.74	100.47	271.42	210.77	199.36	122.69	123.37	38.037	149.94	79.806	2508.8	1320.1	1363.3	601.22	135.64
212464_s_at	2335	FN1	fibronectin 1	26.448	27.259	17.197	26.945	30.48	25.495	42.183	81.327	103.35	16.885	366.11	53.143	16242	58.443	2883.5	2906	165.13
204787_at	11326	VSIG4	V-set and immunoglobuli	233	137.81	120.47	81.189	213.86	154.35	61.94	352.53	358.35	198.53	205.15	68.283	3604.2	370.12	157.55	209.64	455.5
210495_x_at	2335	FN1	fibronectin 1	70.789	23.169	29.882	44.304	59.421	56.337	17.899	52.106	56.731	16.433	698.9	104.41	19697	64.194	4322.9	4511	73.793
221565_s_at	51063	FAM26B	family with sequence sim	314.56	124.06	776.91	70.875	230.96	151.41	104.78	139.07	174.98	129.8	874.44	374.83	2336.4	451.57	899.63	218.93	379.34
219525_at	55244	FLJ10847	solute carrier family 47,	18.184	27.079	14.001	17.424	21.488	30.656	13.634	24.757	22.461	30.17	18.268	31.408	34.706	31.818	1294.5	62.187	37.893
203305_at	2162	F13A1	coagulation factor XIII, A	289.19	154.99	124.88	105.2	313.25	233.58	115.91	479.91	402.89	247.61	199.86	162.12	301.41	267.62	9213.4	463.63	663.21
206682_at	10462	CLEC10A	C-type lectin domain fam	117.66	77.553	54.795	58.318	113.97	116.72	20.433	119.71	111.38	104.15	242.18	51.339	666.23	247.07	10346	234.87	219.09
207328_at	246	ALOX15	arachidonate 15-lipoxyge	10.612	25.339	6.831	6.378	13.36	11.346	9.715	19.972	58.372	10.044	27.642	27.956	26.953	29.212	4539.9	72.346	182.96
204044_at	23475	QPRT	quinolinate phosphoribos	22.06	16.746	54.589	25.22	24.631	16.766	31.21	87.88	56.88	141.4	27.911	18.443	39.471	38.229	3854.3	108.41	77.585
237731_at				40.498	30.122	31.198	25.411	32.529	28.516	36.734	69.953	64.71	44.851	23.433	33.679	57.564	49.195	1113.4	122.32	43.176
203619_s_at	23017	FAIM2	Fas apoptotic inhibitory r	53.577	29.106	14.683	16.222	41.65	21.3	18.821	28.089	25.181	28.634	18.124	15.246	38.708	44.951	1986	47.632	35.496
220301_at	79839	CCDC102B	coiled-coil domain contai	13.112	8.355	4.523	5.864	5.023	8.302	1.015	37.086	9.202	8.124	16.601	9.295	16.732	22.858	224.25	24.244	10.698
201876_at	5445	PON2	paraoxonase 2	153.68	113.96	189.29	208.72	203.37	121.68	97.311	120.96	128.04	130.15	434.92	340.34	276.18	103.43	5310.8	766.1	141.11

207277_at	30835	CD209	CD209 molecule	220.38	169.13	83.777	91.853	207.13	163.27	99.57	313.17	251	187.27	118.57	188.32	365.69	835.46	7460.1	742.95	370.95
222240_s_at	51477	ISY1	myo-inositol 1-phosphate	285.49	212.26	179.91	183.44	292.53	198.33	85.288	65.867	135.68	52.564	172.47	121.04	178.53	181.87	2664.7	426.65	120.38
207278_s_at	30835	CD209	CD209 molecule	106.67	65.52	19.824	18.065	59.958	52.787	21.539	35.029	47.617	24.918	40.149	53.679	90.689	154.74	1321.4	166.3	140.49
201348_at	2878	GPX3	glutathione peroxidase 3	207.97	169.7	117.12	135.49	237.29	182.73	161.49	414.11	438.13	229.3	289.86	166.4	2820.8	773.89	17689	2021.5	666.64
214091_s_at	2878	GPX3	glutathione peroxidase 3	202.77	174.29	157.95	111.93	261.65	236.72	154.17	295.06	301.73	234.74	290.97	235.63	1813.8	724.23	10819	1542.4	612.69
210830_s_at	5445	PON2	paraoxonase 2	142.99	90.515	112.25	122	90.488	64.661	62.882	113.24	149.34	50.288	188.76	161.04	168.75	41.48	3833.2	579.46	69.345
223939_at	56670	SUCNR1	succinate receptor 1	181.08	728.19	164.8	113.29	340.37	277.13	142.74	423.79	260.43	305.45	166.02	133.08	158.29	517.6	4885.7	822.66	343.58
213230_at	30850	CDR2L	cerebellar degeneration-r	14.937	10.283	11.864	17.984	14.459	8.9	13.077	13.102	13.026	10.069	15.938	20.626	54.75	40.053	904.74	77.118	39.601
209183_s_at	11067	C10orf10	chromosome 10 open rea	37.582	61.905	18.124	17.372	28.084	23.813	25.516	43.537	39.406	29.808	24.176	45.143	33.926	63.693	95.817	2692.8	36.858
201564_s_at	6624	FSCN1	fascin homolog 1, actin-b	21.011	314.03	33.576	161.6	16.286	63.516	55.39	54.472	29.717	24.173	81.53	523.84	136.01	184.31	670.32	17366	98.237
218596_at	54662	TBC1D13	TBC1 domain family, mer	76.209	49.734	69.905	50.81	70.849	141.92	114.81	54.299	51.949	51.197	98.944	262.08	96.11	188.52	289.66	5087.5	56.742
237154_at				333.43	340.39	283.22	277.53	237.07	137.2	75.169	147.68	206.61	305.57	150.12	378.01	86.24	121.02	294.11	15782	284.28
205226_at	5157	PDGFRL	platelet-derived growth f	36.427	9.53	14.258	12.599	34.964	16.96	44.181	40.21	22.475	13.408	28.375	53.462	47.721	49.514	53.746	1387.7	20.595
202411_at	3429	IFI27	interferon, alpha-inducib	162.25	912.04	119.25	65.958	75.112	112.25	46.373	50.78	94.163	119.78	134.04	1488.9	210.18	325.75	138.05	17765	151.87
205890_s_at	10537	UBD	ubiquitin D	78.773	668.53	20.265	24.835	25.86	39.337	23.056	114.36	60.898	40.013	74.727	153.21	53.552	33.817	95.649	8820.3	107.24
227970_at	80045	GPR157	G protein-coupled recept	1127.6	936.33	442.99	373.89	792.28	765.69	864.41	672.41	417.83	1021.2	990.63	1117.5	941.2	500.12	390.29	9736.3	730.17
224399_at	80380	PDCD1LG2	programmed cell death 1	37.157	39.408	26.364	31.244	35.784	26.589	26.191	63.203	53.796	59.15	21.387	27.365	24.291	29.793	122.17	1974.3	53.788
209182_s_at	11067	C10orf10	chromosome 10 open rea	86.486	66.895	25.297	43.289	87.619	102.61	23.724	58.954	61.644	63.256	76.594	37.144	76.86	55.657	65.272	1186.5	89.081
211267_at	8820	HESX1	HESX homeobox 1	15.962	20.309	9.002	16.894	20.791	22.89	13.287	26.034	26.317	17.264	13.973	59.359	33.421	25.657	40.231	1691.5	22.801
210933_s_at	6624	FSCN1	fascin homolog 1, actin-b	20.332	566.4	33.336	168.56	71.603	102.41	67.491	10.366	6.506	7.758	126.4	620.29	171.77	344.98	1454.9	16899	52.809
208782_at	11167	FSTL1	follistatin-like 1	192.12	120.83	113.92	109.45	195.78	158.41	118.18	353.01	375.64	162.49	93.606	105.83	192.63	274.55	188.9	4875.7	381.91
44696_at	54662	TBC1D13	TBC1 domain family, mer	624.15	537.35	501.78	399.81	665.39	730.1	446.29	528.19	571.03	405.15	582	689.43	770.72	899.75	1147.7	7639.7	681.05
211083_s_at	9175	MAP3K13	mitogen-activated protei	6.504	4.804	3.886	3.113	5.906	5.464	4.814	8.781	9.781	11.148	9.844	6.678	11.997	17.73	14.728	173.95	11.757
206638_at	3357	HTR2B	5-hydroxytryptamine (se	34.96	28.74	11.051	3.945	26.466	10.004	15.287	25.989	17.253	29.89	44.103	23.668	20.289	20.805	23.311	815.72	23.146
223741_s_at	94015	TTYH2	tweety homolog 2 (Droso	607.39	287.84	245.13	104.92	237.65	192.38	136.59	76.097	105.62	369.95	403.48	548.11	194.02	123.57	283.43	7052.4	203.15
220322_at	56300	IL1F9	interleukin 1 family, men	91.241	91.419	44.993	41.394	72.339	78.498	53.339	171.73	175.77	85.443	75.673	95.566	193.5	13364	96.662	140.58	158.08
207316_at	3036	HAS1	hyaluronan synthase 1	83.836	49.02	39.758	51.693	82.304	62.179	22.661	135.41	153.81	107.37	52.122	23.743	85.531	6520.7	125.32	73.135	144.91
209278_s_at	7980	TFPI2	tissue factor pathway inh	15.299	25.449	6.514	7.562	15.212	135.04	88.061	22.203	21.938	12.314	14.302	264.75	228.32	10802	22.39	274.11	37.116
206569_at	11009	IL24	interleukin 24	85.352	83.076	38.318	38.162	251.23	72.213	118.18	67.58	126.55	22.751	89.089	27.311	98.807	7265	37.723	43.914	230.31
204475_at	4312	MMP1	matrix metallopeptidase	9.388	26.327	2.994	3.6	8.573	16.557	7.346	33.165	45.886	6.634	14.214	3.569	15.962	2464.7	22.158	26.477	38.896
209277_at	7980	TFPI2	tissue factor pathway inh	25.095	20.044	10.747	5.447	35.849	74.723	52.423	70.474	69.994	34.393	31.933	59.772	146.36	3886.6	28.623	142.85	22.053
207442_at	1440	CSF3	colony stimulating factor	18.01	24.088	13.039	11.862	29.41	19.209	19.475	26.511	26.774	20.875	47.925	24.496	27.293	2163.5	30.881	31.705	36.188
205207_at	3569	IL6	interleukin 6 (interferon,	207.34	440.66	94.669	75.292	320.72	1588	284.03	341.25	435.91	145.62	97.655	1336.6	1632.4	34971	204.43	2510.5	240.88
203510_at	4233	MET	met proto-oncogene (hep	11.918	12.065	5.665	56.352	11.912	10.115	35.396	24.648	16.854	32.194	15.715	16.009	360.15	4883.1	27.917	148.52	23.036
229435_at	169792	GLIS3	GLIS family zinc finger 3	51.725	42.231	9.847	76.931	13.202	16.12	14.462	83.659	111.02	64.578	13.486	37.029	185.27	1833	46.519	15.028	127.91
220014_at	51334	PRR16	proline rich 16	11.96	4.882	2.492	0.985	3.236	8.931	22.338	34.53	23.283	19.207	19.619	10.144	72.589	812.71	19.002	17.112	43.945
207852_at	6374	CXCL5	chemokine (C-X-C motif)	31.673	28.613	23.431	24.093	36.395	9.425	17.418	54.262	77.543	38.061	9.621	15.056	175.25	1677.9	40.346	43.681	99.159
207336_at	6660	SOX5	SRY (sex determining reg	9.836	7.861	7.661	6.475	29.154	8.14	11.336	19.539	23.041	16.023	9.393	10.435	26.932	296.21	30.578	32.724	14.187
220655_at	79931	TNIP3	TNFAIP3 interacting prot	30.483	496.06	87	200.55	28.186	19.561	27.081	64.546	62.118	37.863	13.75	34.176	660.08	8318.6	49.416	180.07	35.132
206421_s_at	8710	SERPINB7	serpin peptidase inhibitor	31.11	25.351	15.255	11.639	31.797	20.7	20.595	48.02	44.575	24.085	26.193	269.05	48.317	8770.5	32.96	803.26	45.827
204627_s_at	3690	ITGB3	integrin, beta 3 (platelet	77.937	52.599	25.61	12.752	87.344	100.16	79.679	27.204	28.815	13.054	82.961	102.15	50.385	2003.3	53.605	172.65	112.65
204748_at	5743	PTGS2	prostaglandin-endoperox	75.522	58.612	20.681	22.695	16.161	26.012	21.551	28.498	43.676	25.445	22.052	57.425	434.21	23846	79.633	293.33	2414
211163_s_at	8794	TNFRSF10C	tumor necrosis factor rec	24.745	17.582	11.933	10.705	16.047	12.525	12.11	37.874	17.543	9.775	23.394	13.984	40.419	75.161	46.084	27.389	7494
220005_at	53829	P2RY13	purinergic receptor P2Y,	20.207	15.818	7.568	6.407	29.631	76.257	13.312	70.916	33.945	22.424	15.617	12.05	22.463	29.8	106.12	24.156	7096
203435_s_at	4311	MME	membrane metallo-endo	52.68	35.537	27.734	32.273	47.653	44.56	32.565	106.81	102.32	68.168	45.207	25.599	56.171	108.62	66.398	51.768	5834.4
229967_at	146225	CMTM2	CKLF-like MARVEL transr	404.54	263.4	375.27	305.18	320.72	272.32	165.77	841.78	212.16	494.7	260.95	291.93	161.55	189.59	204.06	184.9	41610
234644_x_at				176.1	142.03	83.535	142.24	49.475	43.257	26.589	45.615	59.935	125.79	95.751	43.935	27.844	33.083	31.309	39.868	11321
206222_at	8794	TNFRSF10C	tumor necrosis factor rec	122.04	82.36	80.849	72.071	101.24	94.444	66.289	143.19	149.05	94.772	69.516	66.842	124.52	98.004	132	127.39	7001.1
204351_at	6286	S100P	S100 calcium binding pro	341.03	96.34	29.987	26.748	230.68	299.56	120.18	69.519	41.807	30.985	35.679	40.597	196.07	287.74	71.192	50.918	21441
207094_at	3577	IL8RA	interleukin 8 receptor, alp	55.571	34.928	30.255	30.118	51.796	46.036	23.438	66.93	68.085	27.242	150.19	36.452	38.777	47.861	34.59	34.741	6702.9
232629_at	60675	PROK2	prokineticin 2	25.175	26.004	158.35	155.29	82.893	49.74	28.545	24.378	16.613	53.691	27.394	7.688	94.419	58.926	11.755	29.657	13698
220945_x_at	54682	MANSC1	MANSC domain containin	75.87	57.915	31.179	33.702	98.717	103.16	34.426	291.74	249.55	97.194	32.466	53.104	75.79	54.837	60.247	61.276	2613.4
229770_at	144423	GLT1D1	glycosyltransferase 1 dor	48.013	28.57	27.015	34.934	109.92	33.965	25.369	54.365	48.216	45.415	97.688	90.763	406.39	290.12	29.735	19.451	12243
207008_at	3579	IL8RB	interleukin 8 receptor, be	48.606	7.934	13.365	4.607	34.921	15.821	19.504	13.723	15.532	19.566	392.19	100.84	111.39	20.749	453.51	83.791	12028
218963_s_at	25984	KRT23	keratin 23 (histone deac	21.721	25.964	13.516	29.433	77.001	62.235	52.891	151.99	138.74	106.63	36.835	49.514	68.24	67.635	90.373	68.849	3796.8
204007_at	2214	FCGR3A	Fc fragment of IgG, low a	420.69	197.38	238.39	130.46	284.33	180.1	91.841	248.53	239.36	162.95	901.02	693.72	1388	330.29	466.68	231.55	32749
203434_s_at	4311	MME	membrane metallo-endo	21.906	20.104	7.349	5.374	33.004	64.683	12.237	32.656	30.344	18.396	18.525	13.907	21.308	93.096	27.296	25.716	2580
220421_at	79908	BTNL8	butyrophilin-like 8	18.42	11.019	9.26	19.771	28.565	22.44	13.582	25.459	26.706	26.866	19.269	15.886	38.278	55.531	50.442	42.432	1195.4
210484_s_at	254896		hypothetical protein MGC	86.434	63.098	71.649	52.289	87.488	50.563	44.979	99.362	61.429	110.7	56.534	90.265	162.63	125.69	139.7	144.21	2915.3
240347_at				1854.5	61.454	48.362	44.29	999.91	57.159	47.999	62.948	64.1	66.571	43.711	36.304	28.181	36.962	26.687	31.636	101.33

227614_at	80201	HKDC1	hexokinase domain conta	442.35	35.677	47.464	21.902	45.899	34.716	31.414	65.151	73.108	71.596	26.961	45.619	23.442	29.542	32.629	24.274	58.015
204760_s_at	9572	NR1D1	nuclear receptor subfami	297.62	19.749	6.99	17.411	66.318	13.655	10.1	11.711	9.278	13.786	10.559	20.107	16.677	28.563	25.355	25.463	33.51
234967_at	3572	IL6ST	interleukin 6 signal trans	500.94	54.356	55.673	34.638	18.455	33.371	45.084	127.48	253.1	93.452	18.648	75.83	17.295	19.91	12.723	20.567	66.204
226342_at	6711	SPTBN1	spectrin, beta, non-eryth	1419.8	51.589	46.397	44.595	332.99	37.121	54.203	108.92	253.63	122.46	40.456	30.066	29.822	33.681	25.029	28.475	88.178
226388_at	6920	TCEA3	transcription elongation f	1084.5	225.21	238.7	62.339	68.575	50.839	46.818	75.369	171.03	152.37	116.74	24.173	42.689	80.724	58.11	61.04	198.9
215262_at				1279.9	295.26	403.78	149.61	92.396	109.06	137.22	125.98	246.18	43.598	125.04	63.024	38.942	29.201	84.617	132.45	136.94
221790_s_at	26119	LDLRAP1	low density lipoprotein re	2504.4	344.2	835.09	62.217	197.35	98.984	173	135.23	159.29	284.72	512.32	232.72	316.47	283.63	570.71	174.09	140.7
230304_at				2596.8	466.59	585.78	390.5	474.34	448.8	270.5	136.42	258	408.3	1183.6	722.03	174.49	97.182	94.599	65.5	229.94
207906_at	3562	IL3	interleukin 3 (colony-stin	11.796	382.21	7.019	134.51	10.679	10.166	9.046	36.347	16.231	9.837	9.531	7.443	16.465	22.998	20.151	21.977	18.31
222974_at	50616	IL22	interleukin 22	51.201	3661.6	26.909	880.39	42.27	53.862	16.571	102.85	70.52	146.28	86.691	40.447	29.994	57.904	25.763	21.935	91.888
227984_at				1219.7	1270.8	138.68	191.49	111.82	123.14	198.43	173.84	193.14	182.04	146.1	153.11	42.787	41.659	142.33	46.938	114.22
232584_at				1125	2341.9	195.23	381.74	356.46	283.78	174.75	205.37	190.32	240.26	149.76	123.63	72.559	162.63	116.99	71.606	208.72
207892_at	959	CD40LG	CD40 ligand (TNF superfa	492.38	838.31	67.853	167.51	104.76	46.611	20.655	64.876	58.402	42.182	40.464	49.649	34.919	30.912	38.454	28.851	88.189
224211_at	50943	FOXP3	forkhead box P3	322.53	1418.7	167.46	635.89	89.541	65.338	48.38	220.77	206.11	146.06	127.15	81.559	32.344	77.866	46.594	46.038	122.99
224801_at				562.06	2564.1	486.91	2284.5	335.08	441.29	350.3	661.08	704.09	614.13	370.94	370.3	183.16	259.95	348.39	267.85	711.33
236341_at	1493	CTLA4	cytotoxic T-lymphocyte-a	1240.2	4434.9	489.94	1618.8	53.729	52.941	16.366	134.12	85.046	151.54	9.094	48.237	37.288	50.691	101.61	64.781	132.91
220485_s_at	55423	SIRPG	signal-regulatory protein	1279.7	344.58	1870.4	377.07	176.03	122.11	36.387	183.64	157.75	97.431	105.48	80.092	107.77	138.53	127.52	151.82	220.38
206804_at	917	CD3G	CD3g molecule, gamma	2246.6	916.04	3622.2	876.21	201.71	87.324	19.905	53.472	55.177	32.644	477.55	288.48	44.127	84.826	45.794	46.494	83.39
206974_at	10663	CXCR6	chemokine (C-X-C motif)	147.04	969.58	1662.6	350.63	135.37	191.12	73.848	262.77	265.17	136.31	406.47	375.57	152.71	177.96	163.36	154.32	292.65
230218_at	3090	HIC1	hypermethylated in canc	37.26	320.67	1359.6	1251.4	43.644	62.872	53.679	62.066	56.536	43.042	190.21	119.43	47.963	42.613	46.522	177.53	73.215
205484_at	27240	SIT1	signaling threshold regul	823.62	686.41	2063	786.65	1305.4	447.22	556.99	658.06	732.74	394.33	199.48	132.05	101.21	123.34	407.85	119.45	174.4
233857_s_at	51676	ASB2	ankyrin repeat and SOCS	380.33	402.91	2482.4	1135.9	242.59	289.52	203.62	242.59	200.29	612.31	274.66	272.28	135.17	143.8	94.268	172.17	376.9
227903_x_at	91978	C19orf20	chromosome 19 open rea	147.3	83.667	652.05	427.54	76.829	56.022	175.17	42.183	46.635	136.02	53.805	54.227	136.66	121.66	142.86	168.85	43.394
209670_at				9535.5	4889.7	11371	4324.1	989.81	734	297.26	509.97	406.85	471.19	786.27	699.43	305.97	565.94	236.82	296.6	844.63
204890_s_at	3932	LCK	lymphocyte-specific prot	4328.6	1960.6	5769.7	2096.1	582.59	401.34	265.26	313.1	297.33	85.982	2360.8	993.94	268.64	389.46	154.42	148.7	415.79
219836_at	79413	ZBED2	zinc finger, BED-type con	153.9	3485.5	158.99	5721.5	244.85	328.8	549.38	314.44	244.52	123.05	130.91	87.84	77.241	139.93	117.45	87.879	257.46
204695_at	993	CDC25A	cell division cycle 25 hon	13.246	24.781	88.757	868.1	16.454	12.278	11.699	27.212	27.494	47.288	12.808	8.661	21.27	35.54	28.316	18.804	23.371
241682_at	151230	KLHL23	kelch-like 23 (Drosophila	60.364	94.954	220.96	1774.3	72.694	58.921	60.413	79.842	48.502	36.977	86.999	91.376	27.185	36.326	19.515	22.162	51.824
225655_at	29128	UHRF1	ubiquitin-like, containing	236.33	1119.1	3816.9	11233	321.11	227.75	796.15	223.23	171.58	1438.7	184.73	95.148	68.223	51.556	36.754	33.832	223.99
242890_at				233.92	290.59	813.35	3551	308.18	279.31	439.72	282.41	256.85	363.93	137.58	95.521	32.602	48.19	34.163	57.902	99.755
223229_at	29089	UBE2T	ubiquitin-conjugating enz	174.69	798.98	1705.3	6581	117.92	255.28	393.67	100.27	84.534	842.79	137.04	110.9	41.216	64.275	62.825	50.933	53.295
220085_at	3070	HELLS	helicase, lymphoid-specif	47.141	87.792	454.04	1303.1	93.846	106.47	136.72	41.992	43.732	61.13	6.926	6.673	16.983	29.67	25.008	24.039	28.993
204558_at	8438	RAD54L	RAD54-like (S. cerevisiae	37.236	27.188	174	529.88	29.891	30.037	23.372	35.105	31.035	17.64	55.063	35.177	56.263	47.656	48.391	46.296	57.866
227350_at	3070	HELLS	helicase, lymphoid-specif	284.84	423.31	1327.9	3798.5	346.27	421.1	591.66	309.23	325.53	424.17	176.76	154	34.591	65.708	34.752	50.621	298.73
205242_at	10563	CXCL13	chemokine (C-X-C motif)	19.513	1354.7	29.476	1874.5	18.396	16.105	190.19	60.868	41.46	23.525	28.792	94.868	25.984	158.1	27.05	242.1	41.323
219000_s_at	79075	DCC1	defective in sister chroma	41.096	98.875	266.43	1054.4	34.983	45.943	45.53	38.164	54.297	115.2	61.206	23.793	101.91	136.67	98.441	95.428	61.939
206975_at	4049	LTA	lymphotoxin alpha (TNF s	36.869	2186	19.704	2896.6	23.214	305.93	19.847	22.388	21.473	14.365	18.138	118.74	20.557	29.577	27.044	28.376	32.132
222504_s_at	10328	COX4NB	COX4 neighbor	27.346	73.761	32.207	695.77	25.677	34.441	63.591	60.775	60.12	98.738	29.093	23.251	36.363	39.324	34.294	22.123	37.046
235401_s_at	84824	FCRLA	Fc receptor-like A	130.31	41.849	42.774	32.087	3782.4	1105.1	2016.2	1131.8	1993.1	118.29	45.306	35.399	33.046	52.991	28.414	16.273	85.514
230877_at	3495		immunoglobulin heavy co	668.07	294.22	50.476	56.156	21387	11410	6419.6	332.54	1278.4	329.1	45.323	42.148	14.306	59.328	16.55	23.564	65.166
227198_at				533.25	92.106	343.14	118.34	9753.7	6992	4487.8	1347.4	2163	17.917	278.65	183.3	30.892	227.31	28.426	22.836	36.823
219667_s_at	55024	BANK1	B-cell scaffold protein wit	131.67	62.309	17.332	13.043	5063.1	3369.7	1609	4849.6	4389	269.4	87.887	93.53	35.155	98.414	81.002	42.046	96.465
217418_x_at	931	MS4A1	membrane-spanning 4-d	756	462.64	129.79	55.3	13482	12327	7914.7	12484	14351	330.25	161.02	229.51	115.45	406.1	130.5	190.71	468.78
243780_at				743.39	815.94	246.9	284.77	15139	11615	7550.3	4127.6	4829.9	1153.1	188.29	83.886	44.602	109.46	38.954	42.475	172.67
210356_x_at	931	MS4A1	membrane-spanning 4-d	909.27	610.61	159.55	92.926	15292	14082	8618.6	14009	16289	294.53	225.68	328.16	265.38	525.55	198.48	283.11	571.83
230983_at	199786	BCNP1	family with sequence sim	1149.9	781.16	583.52	548.37	15403	7850.1	4753.7	3450	6123.6	822.34	702.73	706.12	256.69	301.58	217.31	334.95	1098.2
243968_x_at	115350	FCRL1	Fc receptor-like 1	416.95	132.19	110.29	114.67	10113	3156.3	1094.1	1986.9	2423.5	875.88	108.17	132.07	64.362	69.389	74.8	93.924	289.88
232286_at				415.85	176.56	284.27	153.54	7089.1	3593	3206.7	1259.2	1392.3	207.42	191.43	156.86	97.122	121.18	138.05	110.89	567.87
228599_at	931	MS4A1	membrane-spanning 4-d	291.55	183.21	166.4	186.99	8517.3	10792	5230.7	3721.6	4253.6	312.53	80.829	114.15	101.59	113.37	101.97	103.15	253.83
228592_at	931	MS4A1	membrane-spanning 4-d	1316.4	1018.6	104.4	58.217	33100	35052	20987	23579	26239	395.68	326.13	440.54	128.31	417.2	81.803	158.62	326.6
222891_s_at	53335	BCL11A	B-cell CLL/lymphoma 11	338.58	370.66	69.794	59.023	13141	14169	6339.9	3436.7	3985.5	528.8	278.77	344.03	188.3	1126.3	142.9	935.66	524.55
205933_at	26040	SETBP1	SET binding protein 1	164.27	180.62	200.7	219.58	1157	3083.3	786.02	716.76	599.74	526.88	182.44	205.55	75.283	105.32	347.1	146.98	127.52
219498_s_at	53335	BCL11A	B-cell CLL/lymphoma 11	138.73	133.94	25.675	41.093	2742.6	3364.6	1258.2	1038.8	1261.6	187.64	51.388	86.743	146.78	603.73	84.55	594.52	212.77
217084_at				41.785	67.193	9.726	5.421	163.95	47.628	32.218	45.183	41.792	1278.4	6.244	13.577	15.288	17.852	17.773	16.565	14.048
211642_at				13.19	15.156	7.21	7.64	141.74	45.987	36.33	21.507	51.597	827.93	8.998	9.784	20.519	27.983	23.596	15.285	39.095
217378_x_at				968.69	815.12	23.298	23.718	5042.3	2888.9	2118.1	2838.8	3291.2	34965	22.09	30.102	44.255	58.244	47.213	46.657	53.954
214777_at				443.22	466.97	17.373	12.526	4208.7	2671.2	1577.3	1877	2561.6	34431	25.025	27.54	26.512	58.57	29.03	25.412	43.521
203065_s_at	857	CAV1	caveolin 1, caveolae prot	78.802	62.756	35.597	78.088	88.197	45.911	78.45	119.82	124.39	1721.2	32.745	47.461	43.329	83.562	51.181	114.21	120.86
216557_x_at				677.66	476.06	157.64	134.86	2782.3	1358.7	976.07	737.17	1560.2	21772	156.03	222.68	145.78	228.12	167.6	153.98	378.45
232478_at				21.964	34.023	28.871	23.483	28.206	39.402	43.26	60.684	63.493	39.157	1357.6	1006	18.416	25.441	20.915	20.658	43.551

221417_x_at				15.586	12.486	8.636	8.962	16.528	11.045	13.645	18.64	16.741	10.684	636.77	472.14	16.048	20.789	17.434	18.811	19.978
233743_x_at				67.846	44.061	34.084	28.856	38.968	48.293	33.393	82.363	80.263	80.665	1050.1	856.24	18.334	27.73	21.672	26.124	41.053
230464_at				717.93	282.15	666.48	162.36	414.42	319.18	33.136	75.413	64.085	83.253	10394	9089.6	32.645	56.232	50.011	38.707	78.204
201681_s_at	9231	DLG5	discs, large homolog 5 (I	48.54	24.378	25.433	19.693	44.627	16.9	23.514	39.08	38.174	37.877	593.45	203.75	22.159	47.671	29.067	22.446	35.15
225688_s_at	257068	PLCXD2	phosphatidylinositol-spec	357.59	187.77	433.56	256.29	505.27	522.21	174.05	345.23	277.7	248.14	3883.5	2824.8	57.803	75.66	44.914	112.46	259.01
219383_at	79899	FLJ14213	hypothetical protein FLJ1	180.15	197.03	170.88	145.01	166.91	153.61	34.386	135.85	178.07	56.217	1486.1	742.55	54.253	57.897	48.31	112.77	211.31
204731_at	7049	TGFBR3	transforming growth fact	533.63	343.64	541.31	190.35	192.1	131.58	96.953	183.11	213.56	161.31	4462.7	3660.1	148.41	166.84	82.078	112.41	324.45
205898_at	1524	CX3CR1	chemokine (C-X3-C moti	1329.3	195.08	634.97	42.747	441.76	79.278	23.436	156.29	157.04	89.718	11626	7080.3	846.43	55.954	46.831	65.088	703.38
220646_s_at	51348	KLRF1	killer cell lectin-like recep	728.3	114.8	131.7	24.566	751.36	240.92	38.515	203.31	195.7	100.14	7620.2	5981.3	75.985	74.24	100.25	97.755	204.09
205171_at	5775	PTPN4	protein tyrosine phospha	960.31	792.99	1209.5	415.43	695.24	526.92	334.2	378.6	407.28	491.97	6757.7	6224.2	130.69	179.31	485.75	147.81	541.49
236935_at				424.84	365.3	571.27	223.34	289.89	221.9	158.53	250.08	392.4	343.3	3242.9	2902.9	90.022	88.882	109.14	78.403	328.46
226625_at	7049	TGFBR3	transforming growth fact	2940.3	2267.2	2912	1216.9	588.96	522.78	231.16	741.98	630.24	599.46	14704	12273	167.22	280.79	134	140.35	1264.7
223836_at	83888	KSP37	fibroblast growth factor b	2087.8	79.084	8123.5	2358.6	435.28	230.64	175.08	88.643	54.91	54.863	37240	11588	30.563	76.403	31.688	27.257	521.22
223464_at	114879	OSBPL5	oxysterol binding protein	301	127.76	248.21	171.33	202.29	110.69	69.018	132.79	113.05	114.13	2031.1	2266.5	101.74	59.833	139.28	77.974	127.23
235643_at	219285	SAMD9L	sterile alpha motif domai	768.39	9098.1	1581.8	440.99	1694.1	2391.7	104.07	157.76	419.91	2176.5	2468.3	25077	389.99	535.76	573.04	4217.1	849.73
222816_s_at	54877	ZCCHC2	zinc finger, CCHC domair	2470.2	5750.5	1562.2	2493.9	1912.9	2185.9	1046.7	922.62	816.44	599.11	2777.4	13364	2181.8	2110.6	3070.1	2447.8	1979.3
210865_at	356	FASLG	Fas ligand (TNF superfan	272.33	789.32	739.24	912.05	263.67	181.14	81.324	227.78	206.89	174.67	1767.8	4539.7	170.68	255.05	115.16	146.5	379.82
204070_at	5920	RARRES3	retinoic acid receptor res	2719.8	2664.6	3060.1	235.33	849.11	278.5	127.62	432.41	367.02	1801.9	7967.4	15536	142.96	272	114.99	681.38	388.82
226549_at	388228	SBK1	SH3-binding domain kina	913.96	523.96	775.1	283.02	343.93	279.65	196.79	279.46	191.22	270.74	1362.5	3717.5	45.33	75.229	66.294	84.376	443.53
230036_at	219285	SAMD9L	sterile alpha motif domai	1329.8	9480.5	1860.7	712.66	1880.3	2386.9	353.87	846.09	942.06	2698.5	2070.9	15246	608.57	541.2	934.09	3897.5	1137.3
205237_at	2219	FCN1	ficolin (collagen/fibrinoge	467.99	60.392	78.933	58.575	284.26	339.72	113.46	134.87	123.98	180.89	671.24	314.21	12909	835.39	177.32	267.64	5507.7
215049_x_at	9332	CD163	CD163 molecule	101.82	119.76	79.024	61.356	118.4	74.272	44.964	221.24	226.95	210.04	263.89	77.313	9682.4	8620.1	827.99	239.35	265.16
219890_at	23601	CLEC5A	C-type lectin domain fam	119.11	95.737	92.389	84.429	228.91	150.82	73.627	226.83	219.5	159.9	1117.4	316.18	19857	11344	2299.7	896.82	347.84
203645_s_at	9332	CD163	CD163 molecule	71.858	85.886	38.251	24.845	68.466	53.077	31.555	174.52	279.05	140.63	241.85	36.597	8883.9	7051.5	908.66	212.78	175.95
223204_at	51313	C4orf18	chromosome 4 open reac	156.34	93.035	118.44	123.52	130.26	140.87	68.801	294.18	302.38	218.98	437.88	202.42	4948.7	822.28	4023.9	569.82	309.26
204150_at	23166	STAB1	stabilin 1	128.66	107.3	93.651	72.915	83.267	94.68	113.24	128.71	106.57	64	600.3	293.96	10839	1936.2	8595.5	1317.1	174.1
223567_at	10501	SEMA6B	sema domain, transmem	33.318	21.347	24.505	33.317	76.208	44.16	22.369	63.408	40.779	118.86	107.53	73.612	1094.7	910.27	52.127	143.82	30.585
201506_at	7045	TGFBI	transforming growth fact	314.89	110.94	91.258	36.355	79.571	101.04	43.168	62.25	132.31	102.34	3343	793.16	31162	3217	25407	5132.9	103.22
204392_at	8536	CAMK1	calcium/calmodulin-depe	231.77	200.59	264.45	485.72	194.55	167.43	176.85	157.92	185.87	191.37	336.18	168.74	4099.8	349.29	3162	432.58	573.02
38487_at	23166	STAB1	stabilin 1	161.92	111.78	150.14	170.8	190.98	219.39	144.43	301.11	260.52	81.386	705.38	383.12	13007	3057.7	9961.8	2083.4	329.11
213119_at	206358	SLC36A1	solute carrier family 36 (	69.352	112.18	167.49	162.58	136.7	242.86	134.87	81.805	73.633	100.04	249.87	161.79	2534.6	715.27	1603.8	653.16	330.92
215784_at	913	CD1E	CD1e molecule	23.446	31.112	24.51	19.626	10.493	18.039	17.494	38.018	79.575	21.279	40.886	10.775	97.286	121.26	16075	5709.7	69.112
202953_at	713	C1QB	complement component	57.155	68.223	38.545	24.608	93.249	62.42	33.698	50.778	36.277	18.398	77.011	102.19	182.48	146.48	10592	7856.7	41.177
218232_at	712	C1QA	complement component	62.558	24.101	23.867	20.628	32.379	49.396	11.998	83.199	44.593	74.948	50.142	86.203	184.27	108.09	7282.4	2928.5	76.151
206749_at	910	CD1B	CD1b molecule	116.44	64.685	43.037	23.273	75.038	76.53	60.891	184.23	121.28	202.85	106.28	55.634	90.609	383.87	17114	4264.6	170.68
208592_s_at	913	CD1E	CD1e molecule	76.574	73.689	34.628	34.141	101.33	67.955	48.996	74.338	72.827	86.424	115.69	80.099	81.505	94.16	6752.7	1417.6	118.17
225353_s_at	714	C1QC	complement component	110.65	140.74	27.702	61.741	34.068	71.675	33.642	118.86	56.562	125.48	139.21	130.21	495.99	88.076	12507	11043	156.99
204518_s_at	5480	PPIC	peptidylprolyl isomerase	47.172	48.238	37.09	16.31	46.384	27.873	72.802	76.562	100.23	28.991	26.824	12.981	32.336	55.873	707.73	310.34	39.174
206120_at	945	CD33	CD33 molecule	23.86	26.068	11.41	9.258	25.716	20.375	14.645	37.029	35.662	17.374	19.578	19.477	679.76	121.04	1593.4	133.38	60.619
210325_at	909	CD1A	CD1a molecule	334.06	215.3	239.04	192.86	595.37	330.27	283.03	765.57	729.57	334.33	236.08	255.17	374.32	400.57	17758	3408.6	715.59
217757_at	2	A2M	alpha-2-macroglobulin	56.154	90.243	91.964	68.09	88.213	61.413	70.048	110.28	217.55	116.79	212.65	114.82	533.47	194.51	14937	15010	110.11
206407_s_at	6357	CCL13	chemokine (C-C motif) li	103.8	102.11	70.412	68.673	165.5	111.1	53.946	134.15	164.72	100.83	157.64	289.58	242.42	455.08	6434.5	11487	174.82
213415_at	1193	CLIC2	chloride intracellular chai	19.734	13.509	4.991	7.239	27.693	27.378	16.056	17.633	17.894	9.958	115.54	209.36	190.72	234.49	3590.7	8375.9	22.283
203680_at	5577	PRKAR2B	protein kinase, cAMP-dep	109.78	78.126	61.402	92.489	117.09	64.795	78.53	96.114	111.03	96.123	108.68	84.149	166.4	144.73	669.88	3171.9	167.26
219519_s_at	6614	SIGLEC1	sialic acid binding Ig-like	23.821	16.503	13.074	11.874	18.704	14.555	17.02	27.273	27.555	13.05	29.371	89.235	275.86	294.87	710.24	6005.1	49.332
220049_s_at	80380	PDCD1LG2	programmed cell death 1	16.213	14.334	24.966	7.891	14.958	12.758	13.968	20.981	22.152	13.788	33.651	23.536	50.538	60.569	174.05	1520.5	26.35
205686_s_at	942	CD86	CD86 molecule	183.62	162.09	106.74	90.275	293.85	267.82	183.33	618.49	400.78	281.29	137.07	318.48	630.29	242.14	2070.3	8560	203.25
202357_s_at	629	CFB	complement factor B	136.58	132.94	46.834	56.07	131.35	83.048	43.087	129.28	79.264	59.938	110.84	127.86	139.77	1128.5	252.42	4969.9	187.81
204614_at	5055	SERPINB2	serpin peptidase inhibitoi	104.82	108.06	56.517	32.963	71.147	96.217	41.219	113.71	115.17	71.027	83.1	95.581	4287.5	24461	98.613	352.89	181.87
219093_at	55022	FLJ20701	phosphotyrosine interact	68.932	25.883	27.311	36.14	27.062	43.915	33.14	85.735	108.6	45.643	154.69	54.538	7487.2	11205	34.221	105.68	78.269
207850_at	2921	CXCL3	chemokine (C-X-C motif)	24.788	25.074	21.049	26.677	53.966	18.063	38.137	56.646	50.331	87.434	63.616	22.828	3914.7	27356	131.59	522.63	73.882
215101_s_at	6374	CXCL5	chemokine (C-X-C motif)	32.322	5.255	14.219	3.561	33.529	12.421	21.868	49.028	37.239	15.912	20.307	12.939	4063.1	33167	20.269	445.88	102.86
204470_at	2919	CXCL1	chemokine (C-X-C motif)	102.29	72.235	49.323	38.146	109.13	94.808	78.084	156.84	130.59	91.55	86.887	118.64	6214.2	35585	100.46	1191.3	1368.5
210118_s_at	3552	IL1A	interleukin 1, alpha	88.623	145.24	90.422	35.318	155.24	63.8	81.281	287.8	220.03	111.54	112.61	77.232	2599.8	22595	213.69	660.82	323.06
217767_at	718	C3	complement component	59.883	26.757	34.401	17.188	28.973	26.25	17.639	27.042	25.763	39.066	62.908	45.944	5647.8	8096.8	246.51	282.92	49.628
221463_at	6369	CCL24	chemokine (C-C motif) li	44.054	21.628	24.646	14.316	57.382	49.296	26.492	25.721	26.113	19.318	235.72	198.21	9587.6	13839	113.99	365.61	68.75
210511_s_at	3624	INHBA	inhibin, beta A	16.237	135.48	7.304	7.813	26.306	14.216	20.578	20.333	18.133	8.488	20.287	104.69	536.09	11449	171.22	4927.5	19.921
205922_at	8875	VNN2	vanin 2	269.9	77.17	513.82	12.6	355.24	189.21	167.27	287.16	314.52	168.83	499.61	320.66	223.25	4735.8	44.71	72.248	19069
203591_s_at	1441	CSF3R	colony stimulating factor	106.53	44.825	45.942	38.679	176.05	82.909	41.943	74.399	38.012	29.283	24.289	44.759	819.72	100.75	190.43	109.83	10132
223809_at	64407	RGS18	regulator of G-protein sig	348.74	282.09	132.72	112.99	257.38	151.54	93.752	264.46	229.04	186.15	315.04	200.03	374.78	94.765	1421.8	103.53	10444
